# Synaptonemal & CO analyzer: A tool for synaptonemal complex and crossover analysis in immunofluorescence images

**DOI:** 10.3389/fcell.2023.1005145

**Published:** 2023-01-19

**Authors:** Joaquim Soriano, Angela Belmonte-Tebar, Elena de la Casa-Esperon

**Affiliations:** ^1^ Centro Regional de Investigaciones Biomédicas (CRIB), Universidad de Castilla-La Mancha, Albacete, Spain; ^2^ Biology of Cell Growth, Differentiation and Activation Group, Department of Inorganic and Organic Chemistry and Biochemistry, School of Pharmacy, Universidad de Castilla-La Mancha and IDISCAM, Albacete, Spain

**Keywords:** meiotic recombination, crossover, synaptonemal complex, image analysis, ImageJ, Fiji, open-source software

## Abstract

During the formation of ova and sperm, homologous chromosomes get physically attached through the synaptonemal complex and exchange DNA at crossover sites by a process known as meiotic recombination. Chromosomes that do not recombine or have anomalous crossover distributions often separate poorly during the subsequent cell division and end up in abnormal numbers in ova or sperm, which can lead to miscarriage or developmental defects. Crossover numbers and distribution along the synaptonemal complex can be visualized by immunofluorescent microscopy. However, manual analysis of large numbers of cells is very time-consuming and a major bottleneck for recombination studies. Some image analysis tools have been created to overcome this situation, but they are not readily available, do not provide synaptonemal complex data, or do not tackle common experimental difficulties, such as overlapping chromosomes. To overcome these limitations, we have created and validated an open-source ImageJ macro routine that facilitates and speeds up the crossover and synaptonemal complex analyses in mouse chromosome spreads, as well as in other vertebrate species. It is free, easy to use and fulfills the recommendations for enhancing rigor and reproducibility in biomedical studies.

## Introduction

**FIGURE 1:**
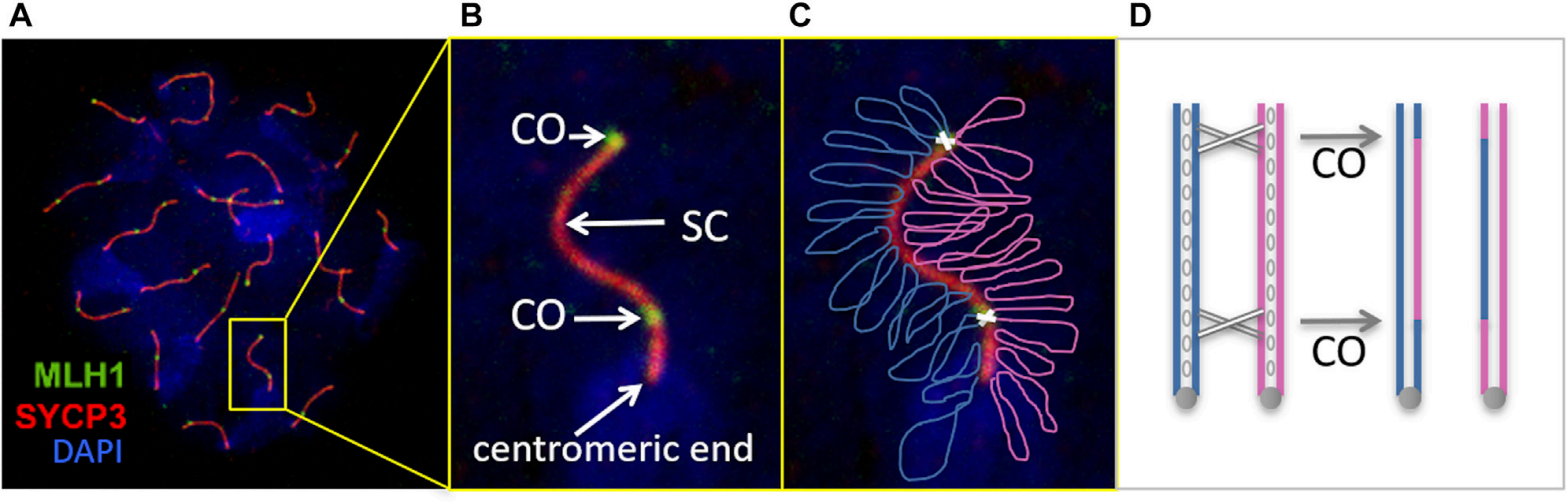
Male meiosis recombination visualized by immunofluorescence of a mouse pachytene-stage nucleus spread. **(A, B)** The spermatocyte was immunostained with antibodies against MLH1 in order to identify the crossover sites (CO) between paired homologous chromosomes. These are joined together through the synaptonemal complex (SC), visualized with antibodies against the protein SYCP3, one of SC components. Chromosomes are not fully condensed at this stage, but DNA staining with DAPI allows to identify each nucleus spread. Consequently, centromeres are not visible yet as a chromosomes constrictions, but can be located with specific probes or by brighter DAPI staining (as indicated in B; notice that mouse centromeres are not central, but distal). **(C)** Simplified representation over the previous image of the two homologous chromosomes (blue and magenta) with two COs (white crosses). These result in the mutual exchange of genetic material between homologous chromosomes, as schematized in **(D)** (each chromosome represented by two identical DNA copies (sister chromatids, resulting from previous DNA replication) joined by the centromere (grey circles) as well as other proteins (cohesins, blank ovals)).

Ova and sperm are formed through a special type of cell division known as meiosis, in which homologous chromosomes exchange genetic information. This process, known as meiotic recombination, requires programmed, developmentally regulated double strand breaks (DSBs) initiating pairing of homologous chromosomes and assembly of a zipper-like multiprotein structure between them (the synaptonemal complex, SC); then, crossovers (COs) between paired chromosomes result in the mutual exchange of genetic material at the pachytene meiosis stage (Figure 1). COs are important for subsequent chromosome segregation during the first meiotic division: those that do not recombine often appear in abnormal numbers in ova, sperm and the resulting embryos, leading to infertility, miscarriage and birth defects (Hassold and Hunt, 2001). Therefore, crossovers not only generate genetic diversity, but are also required for proper chromosome segregation in many sexually reproducing organisms. Hence, meiotic recombination studies are of paramount interest in farming, stockbreeding and human fertility and health (Notter, 1999; Hassold and Hunt, 2001; Handel and Schimenti, 2010; Henderson and Bomblies, 2021).

Immunofluorescence of chromosome spreads of pachytene-stage oocytes or spermatocytes (ova and sperm precursors) has become the most common approach to study meiotic recombination in animals (Baker et al., 1996; Anderson et al., 1999; de Boer et al., 2009; Cole et al., 2012; Imai et al., 2021). For instance, a typical protocol for mouse and other vertebrates’ recombination studies uses antibodies against the mismatch repair protein MLH1 to identify CO sites, antibodies against SYCP3 to label SCs, and DAPI to stain DNA and delimit the nuclei, since chromosomes are not fully condensed and discernible at pachytene stage (Figure 1). Since MLH1 signal is usually weak, in order to tell apart false positives, only MLH1 foci over SYCP3 labeling are considered true COs. If necessary, the centromeric regions of the chromosomes can be recognized with specific labels (CREST serum) or by a more intense DAPI staining (Anderson et al., 1999; Froenicke et al., 2002; Segura et al., 2013) (Figure 1).

The frequency and distribution of COs along SCs are characteristic of each species, though differences may occur between the sexes. Usually, there is at least one CO per SC [the “obligate” crossover required for proper chromosome segregation (Mather, 1937)]. The maximum number depends on the length of the chromosome and the degree of interference between COs, a phenomenon by which the occurrence of one CO interferes with the appearance of a second one nearby (Sturtevant, 1915; Muller, 1916; Sym and Roeder, 1994; Kleckner, 2006). Consequently, high CO frequencies have been associated with either long SCs or weak interference (Anderson et al., 1999; de Boer et al., 2009). Other factor that affects the CO distribution in many species is the CO suppression around the centromeres -chromosome constrictions that play important roles during cell division. For instance, in mouse spermatocytes centromeres are located at one extreme of the chromosomes and, consequently, crossovers accumulate towards the opposite end (Anderson et al., 1999) (Figure 1). This distribution is biologically relevant, because COs too close to the centromeres lead to abnormal chromosome disjunction during cell division (Koehler et al., 1996; Lamb et al., 1996; Hassold and Hunt, 2001).

The relevant data for recombination studies that can be extracted from immunostained pachytene-stage cells are: 1) number of COs per cell and per individual SC; 2) number of SCs per cell and length of each one; 3) distribution of COs on each individual SC relative to, for instance, the centromere. This requires unambiguous identification of the SCs (as they often overlap) and COs on them, as well as the location of the centromeres. In mouse and other eutherian mammals, X and Y chromosomes behave differently than the rest (autosomes) because they only pair and recombine through a small (pseudoautosomal) region; for this reason, they are excluded from many recombination studies in males (Baier et al., 2014; Dumont, 2017).

While MLH1 immunodetection has become a common procedure for many recombination studies, manual COs and SCs image analysis can be very time-consuming and, hence, constitute a major bottleneck. The analysis is also prone to a certain degree of subjectivity, a problem that has been circumvented in some studies by duplicating the image scoring by two independent observers (Baier et al., 2014; Vrooman et al., 2015). Image analysis automation could solve these problems by fastening the procedure and applying objective detection algorithms. A common approach is to develop custom-made software solutions. Regrettably, they usually do not find widespread usage outside the originating lab (Swedlow and Eliceiri, 2009; Prevedello and Khorasani, 2012; Karopka et al., 2014) due to what some authors call a lack of usability (Carpenter et al., 2012), rigor and reproducibility (Brito et al., 2020). In order to facilitate recombination analyses to a broad research community, software should be easy to access and use, well documented and supported (Carpenter et al., 2012; Brito et al., 2020).

Indeed, a few tools have been developed for SC analysis (de Boer et al., 2009; Milano et al., 2019; Peterson et al., 2019; Wang et al., 2019), but none of them are able to extract all the aforementioned meaningful data from recombination studies while fulfilling the requirements for software usability and reproducibility (Carpenter et al., 2012; Brito et al., 2020). The software quoted in de Boer et al. (2009) (Object Image and MicroMeasure) are no longer available in the cited websites. They are intended for SC measurement only and, even though the authors cite the possibility of using a specific macro to measure CO sites and SC length, regrettably it has not been published and is only available upon demand. The macros published in Milano et al. (2019); Wang et al. (2019) do not consider CO nor centromere analysis, and while no information on how to implement the former is available, the latter relies on a specific Python 3 package that is not accessible to users without programming skills. CO detection software based on MLH1 foci detection have also been developed (Martin et al., 2014; Enguita-Marruedo et al., 2019) however, they do not analyze SCs and, therefore, are unable to discriminate true COs from artifacts. Finally, the application developed by Peterson et al. (2019) undertakes a different approach by analyzing large numbers of images in an unsupervised manner while relying in post-processing analyses to remove undesired outcomes. This results in relevant data losses, because overlapping SCs are manually eliminated and sex chromosomes are excluded by size filtering along with other long chromosomes, restricting the analysis to short chromosomes. Overall, this approach is only useful in very large experimental datasets, but implies manual curation of thousands of images (Peterson et al., 2019). Moreover, this solution relies on a software, CyVerse, that is not very common among image analyzers and is only available upon demand.

We decided to develop our own application to study meiotic recombination and to share our efforts by meeting the requirements stated for free software distribution in Carpenter et al. (2012) and the recommendations of Brito et al. (2020) to enhance rigor and reproducibility in biomedical research. Hence, we chose to develop an open-source application as an extension of ImageJ/FIJI (Schindelin et al., 2012; Schneider et al., 2012) because it is the most popular, open-source software for bioimage analysis with a large and interactive user’s community (ImageJ, n.d.; FIJI, n.d.; ImageJ Information and Documentation Portal, n.d.; FIJI Software, n.d.; ImageJ Conferences, n.d.). Therefore, our software has the potential of being easily improved or adapted by other ImageJ/FIJI users to the particular needs of their recombination studies.

## Materials and methods

### Hardware and software characteristics

The software was written in ImageJ’s script language on FIJI, using ImageJ 1.53c. On a PC with Windows 10 operative system working on an Intel Core i5-4200 CPU @ 1.60 GHz 2.30 GHz and 4.00 GB RAM. Gabriel Landini’s Morphology package (Landini, 2008) and the Bio-Formats importer plugin (Linkert et al., 2010) are required for the software to work.

### Validation data sets

Software’s efficiency and accuracy were validated on images from mouse pachytene spermatocytes immunostained with antibodies against MLH1 and SYCP3, counterstained with DAPI and captured under a confocal microscope as previously described (Anderson et al., 1999; de Boer et al., 2009; Milano et al., 2019; Belmonte-Tebar et al., 2022).

Software’s flexibility and applicability were validated on pachytene-stage nuclei images from other species, antibodies and capturing methods (Supplementary Figure S1). Images labeled with MLH1 and SYCP3 antibodies were obtained with protocols similar to ours; some lacked DAPI staining or used human calcinosis, Raynaud’s phenomenon, oesophageal dysfunction, sclerodactyly and telangiectasia (CREST) serum for centromere detection (Segura et al., 2013). They were generously donated as follows: wild-captured house mice *Mus musculus domesticus* with standard karyotype and with Robertsonian translocations (courtesy of Cristina Marin and Aurora Ruiz-Herrera (Vara et al., 2021)); Matthey’s mouse (*Mus matheyi*, courtesy of Jesus Page (Universidad Autonoma de Madrid (UMA), Spain) and Frederic Veyrunes (Universite Montpellier, France); mongolian gerbil (*Meriones unguiculatus,* also of Jesus Page); zebrafish (*Danio rerio*, courtesy of Yukiko Imai, National Institute of Genetics, Japan); chicken (*Gallus gallus* (del Priore and Pigozzi, 2020)) and duck (*Anas platyrhynchos*; both bird images were obtained with antibodies against SMC3 instead of SYCP3 for SC labeling and donated by Maria Ines Pigozzi, Instituto de Investigaciones Biomedicas, Universidad de Buenos Aires-CONICET, Argentina). Generous donations were also *Mus musculus* images stained with antibodies against RAD51 (Jesus Page, UMA) and RPA2 (Parijat Chakraborty and Francesca Cole, The University of Texas MD Anderson Cancer Center, United States).

### Software development and validation processes

**FIGURE 2:**
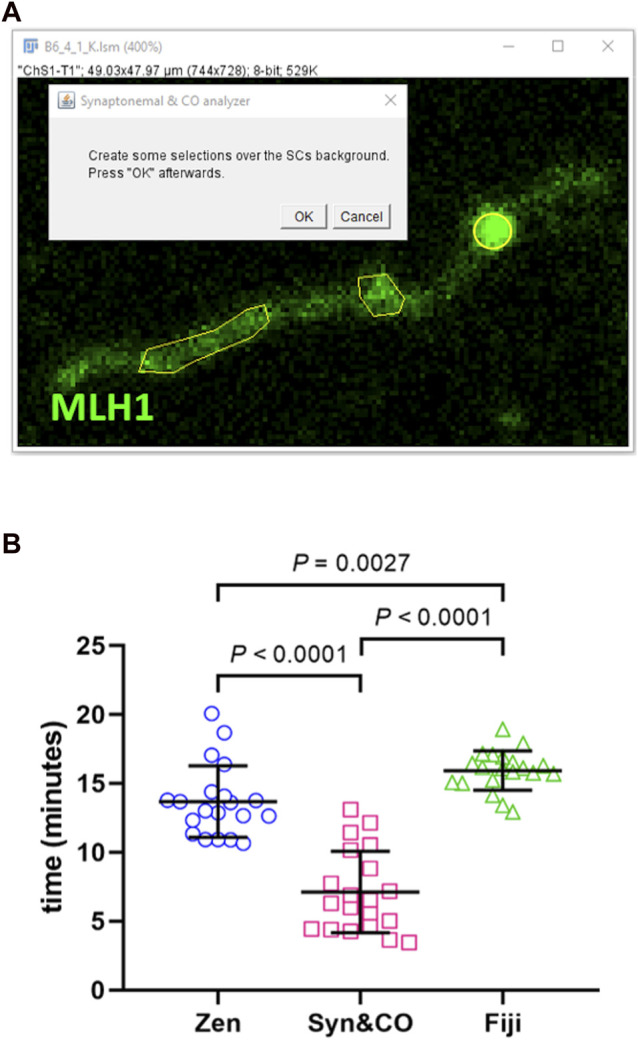
Synaptonemal & CO Analyzer facilitates recombination analysis. **(A)** The macro reduces a complex analysis to easy steps. For instance, the user is asked to draw a few selections (yellow polyhedrons) to launch an automated algorithm to detect COs (yellow circle). **(B)** Synaptonemal & CO Analyzer is, on average, two times faster than manual methods. Synaptonemal & CO Analyzer (Syn&CO) performance compared to FIJI and Zen Lite manual analysis. The same blinded images (n = 20) were analyzed by each method. The results were analyzed by generalized linear models and Bonferroni *post hoc* test. Bars and whiskers represent means and SDs.

Image analysis using Synaptonemal & CO Analyzer is a semi-automated process. Semi-automated SC identification relies on automatically subtracting background using a gaussian filter and a rolling ball algorithm (Sternberg, 1983). The user setting an intensity threshold is the only manual step needed. Afterwards, some binary operations are automatically performed: a reconstruction to get rid of small objects, a closing and an opening to smooth surfaces, and finally getting a SCs’ skeleton. Semi-automatic CO and centromere detection is based on an intensity and size algorithm: whatever is brighter than the background and bigger than pixels is selected. The user needs to determine the background by creating a selection over it (Figure 2).

Exact details on SCs, COs, and centromeres’ detection algorithms can be found in the macro source code by looking for “function SC_analysis,” “function CO_analysis” and “function centromere_analysis,” respectively. Although they work well with most of the tested images, isolating detection algorithms into functions eases adapting detection to new image characteristics. In order to do so, users only need to change the function’s code by a new one. This task has been eased to users with no image analysis background by providing two extra macros (skeletonize_SC_macro_ recorder.ijm,foci_detection_macro_recorder.ijm) that generate detection code, (Supplementary Video tutorial 2 and User Manual). Modifying objects detection algorithms avoids manual steps (such as setting an intensity level on each analyzed image) making the macro more automated. Macro source codes are available at https://github.com/joaquim-soriano/Synaptonemal-and-CO-analyzer.

The macro was developed following a two-step procedure. First, we developed an initial version on mice pachytene spreads (as mentioned above), that was validated for efficiency and accuracy. Second, we adapted the macro to ease work on other species and labels. In the first phase, the software development team consisted of an image analyst, a project manager and a beta tester. The project manager, a meiosis expert, determined the software requirements for recombination studies. The image analyst devised the algorithm and wrote the code, and the beta tester checked the resulting script on a set of standard images. Errors detected and new requirements were reported to the image analyst that fixed the former and implemented the latter. Software’s first version was released after no further requirements were found and results were consistent with those obtained from manual analyses of a set of standard images. These consisted of a representative sample of 20 images of 20 immunostained mouse spermatocytes of an ongoing research project. Each image was manually analyzed with FIJI and with the software previously employed in our laboratory, Zen lite (Zeiss, Oberkochen, Germany), as well as with our semi-automated software. Images were randomized and the identities were blinded and coded differently for each of the three analyses until all were completed in order to avoid bias. The beta tester was previously trained on the use of each analysis method with an independent set of images. Data were obtained on a PC running Windows 10 operative system on an Intel Core i7-7500U @ 2.70 GHz 2.90 GHz and 8.00 GB RAM. Total SC length, number of COs per cell (excluding X and Y chromosomes) and duration of the analysis were compared between the three methods in order to determine the script’s accuracy and efficiency. Results were analyzed by generalized linear models (GLM repeated measures) and Bonferroni *post hoc* test with SPSS software (NIH, Bethesda, MA, United States).

In a second phase, software’s first released version was checked against a diversity of images (Supplementary Figure S1) resulting on a second macro version that opens different image formats, works on centromere-specific labeling (e.g., with CREST serum) and provides means to easily adapting the macro to detect objects under different image conditions.

## Results and discussion

### Software analysis process and outcomes

Image analysis using Synaptonemal & CO Analyzer is a semi-automated process. Once launched, a set of windows ask the user to perform easy tasks (Figure 2 and Supplementary Material: Video tutorial 1 and User Manual) until the software gathers all needed data to automatically perform the analysis. Once done, SCs, COs and centromeres are analyzed sequentially (Supplementary Figure S2) following a similar process (Supplementary Figure S3). Basically, the user decides whether to detect COs or centromeres manually (if the image quality is too low) or introduce parameters for an automated analysis (SC automated detection is always done by default), some checking steps are then performed that might need further user interaction (for example, replacing a CO that does not lay over a SC or isolating overlapping SCs) before the analysis is complete.

**FIGURE 3:**
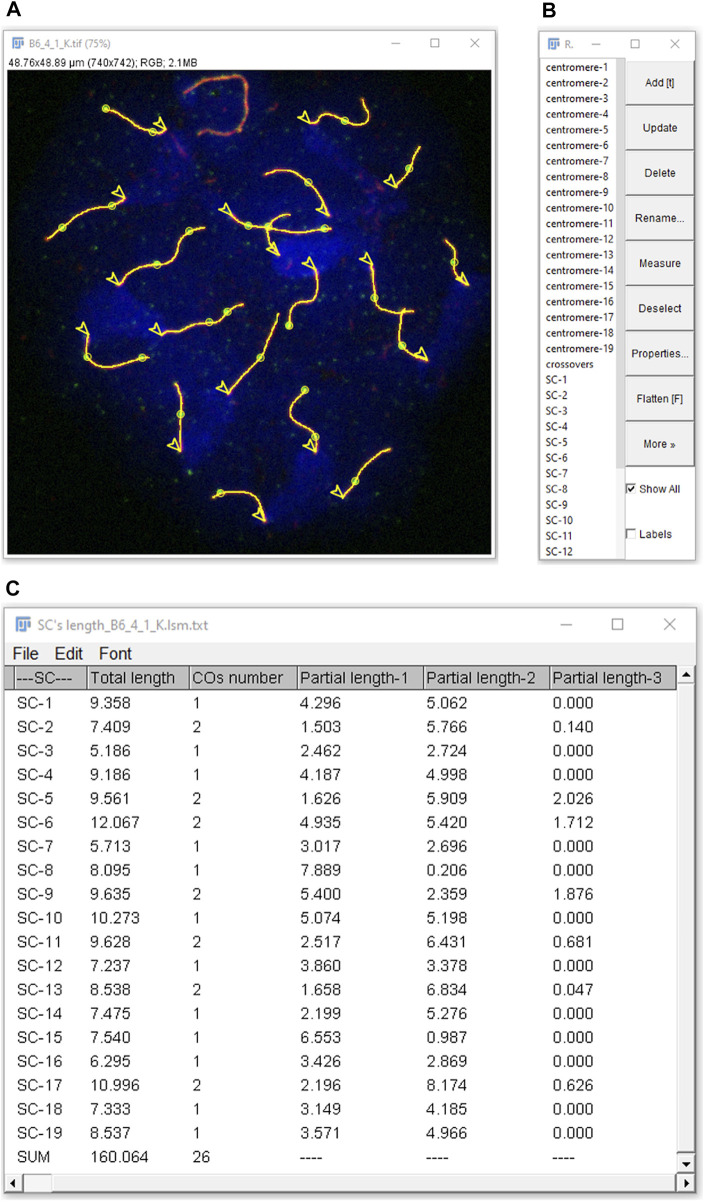
Analysis results as displayed in FIJI. Detected elements (SCs, COs, centromeres and nucleus) can be selected in the ROI Manager **(B)** to be highlighted in the RGB image **(A)**. In this example, selecting “show all” displays everything (lines: SCs; circles: COs; arrow heads: centromeres, from which SC measurements start), except on the XY chromosomes (on the top), which were excluded from the analysis during the nucleus selection step. **(C)** Results are either global (sum of COs number and of total length of the SCs per nucleus) or SC-related: COs number per SC, total length of each SC and partial lengths from one SC end (centromeric, if selected as in the figure) to closest CO (partial length-1), between consecutive COs (if more than one) and between opposite SC end to closest CO (partial length-2, *etc.*).

Synaptonemal & CO Analyzer obtains the following data from pachytene-stage nuclei images: 1) SC length of each chromosome, 2) sum of the length of all the SC per cell, 3) number of COs per SC (i.e., number of COs between each pair of homologous chromosomes), 4) total number of COs per cell and 5) CO location along each SC (Figure 3). CO distances are measured starting from one end, with the option of automatically selecting the centromeric end when discernible. If centromeres’ detection is based upon centromere labels, the position of each centromere will be also delivered, as well as lengths between SCs ends and COs relative to centromere position and the number of COs per chromosome arm. The application also allows for excluding sex chromosomes, thus restricting the analysis to autosomal chromosomes (Baier et al., 2014; Dumont, 2017). Moreover, the macro solves frequent practical issues by providing tools, for instance, to analyze overlapping SCs.

### Software requirements and limitations

The macro assumes that SCs are linear, COs and centromeres lay over SCs, and that the number of centromeres per SC is either one or none. These criteria allow to discriminate true from background foci and are optimal for the analysis of pachytene-stage cells, but not for other stages when SCs are not fully formed. The macro does not impose limits to image quality; however, poor stained materials and ill captured images limit results’ quality and increase analysis’ time. According to our experience, confocal microscopes deliver better results than conventional fluorescence ones, plan apochromatic objectives and close-emitting fluorochromes avoid signal mismatch due to lack of color aberration correction and meeting the Nyquist theorem assures optimal image resolution (Sanderson, 2020).

The macro relies on the Bio-Formats importer plugin to open many dozens of proprietary life science image formats (Linkert et al., 2010) besides the standard ones (tiff, jpeg, *etc.*). Up to seven channel images are supported; however, the macro is designed to analyze 2D images only. Users willing to analyze images with different planes need to collapse them on a single one. This might introduce changes on SCs’ length and shape or cause too many SC overlaps as for the application to efficiently discriminate them. Therefore, the tool is not suitable for immunostained intact nuclei such as those employed in *C. elegans* recombination studies (Garcia-Muse, 2021). In other cases, the user should inspect the images to tell whether these changes occur and are relevant for the desired analysis. In contrast, 2D images with good chromosome spreads minimize the amount of SC overlapping and the macro analysis time and are, therefore, recommended.

### Synaptonemal & CO analyzer provides reliable and fast CO and SC data

When comparing our new application with manual analyses using FIJI or Zen lite, similar results both in number of COs and in total autosomal SC length per cell (the sum of the length of all the SC, excluding the X and Y chromosomes) were obtained (*p* = 0.308 and *p* = 0.147, respectively, GLM). This indicates that the method of choice has no significant effect in the results, thus validating our application. However, when the duration of the complete analysis of COs and SCs was compared, a significant effect of the software of choice was observed (*p* < 0.0001, GLM). Bonferroni *post hoc* analyses revealed that Synaptonemal & CO Analyzer (7.1 ± 3.0 min, mean ± SD) is significantly faster (about two times quicker) than the rest (Zen lite: 13.7 ± 2.6 min, and FIJI: 15.9 ± 1.4 min) (Figure 2). The analysis time is variable depending on the quality of the image and the manual CO and SC corrections required; nevertheless, differences are clearly significant (Figure 2). Given the accuracy and speed of Synaptonemal & CO Analyzer, we have already successfully used it in a study performed by our group (Belmonte-Tebar et al., 2022)

### Applicability: Synaptonemal & CO analyzer for the analysis of images immunostained with various antibodies and from diverse vertebrate species

**FIGURE 4:**
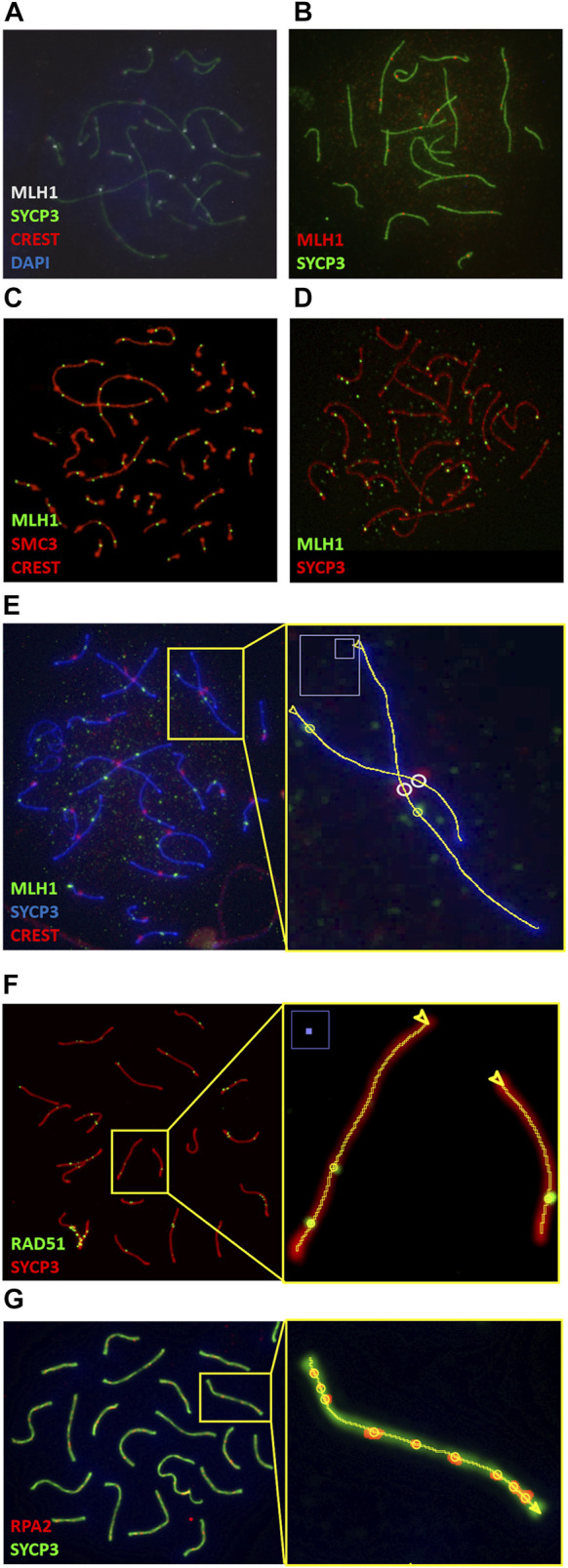
Synaptonemal & CO Analyzer is a versatile tool for the analysis of immunostained pachytene cells. Examples of image analyses from diverse vertebrates: **(A)** wild-captured house mice (*Mus musculus domesticus)* with Robertsonian translocations [courtesy of Cristina Marín and Aurora Ruiz-Herrera (Vara et al., 2021)]. **(B)** Matthey’s mouse (*Mus matheyi*, courtesy of Jesus Page and Frederic Veyrunes); **(C)** chicken (*Gallus gallus*, courtesy of María Inés Pigozzi (del Priore and Pigozzi, 2020)); **(D)** Zebrafish (*Danio rerio*, courtesy of Yukiko Imai); **(E)** mongolian gerbil (*Meriones unguiculatus*, courtesy of Jesus Page), **(F, G)** nuclei from mouse inbred strains (*Mus musculus*) labeled with antibodies against RPA2 and RAD51 (courtesy of Parijat Chakraborty and Francesca Cole, and Jesus Page, respectively). **(E, F)** show magnified views of the elements detected by the macro in sections on the right. The software identifies SCs (lines), COs (yellow circles) and, when applicable, centromeres (white circles); arrow heads indicate the SC end from which SC measurements start. It performs well with diverse fluorochromes, central or distal centromeres stained with DAPI or CREST, and diverse antibodies for CO and SC identification.

Immunostaining of pachytene-stage chromosome spreads with MLH1 and SYCP3 antibodies and DAPI is a common technique for the study of recombination in diverse species. Our application is capable of successfully analyze such images in many vertebrates, including mammals with diverse karyotypes, birds and fish (Figure 4 and Supplementary Figure S1).

Recombination studies are also performed with other immunostaining methods. COs are one of the results of the repair of the hundreds of DSBs that occur at the beginning of meiosis. The progression of recombination intermediates can be examined by labeling proteins other than MLH1 (Hunter, 2015; Zickler and Kleckner, 2015; Gray and Cohen, 2016). The analysis of pachytene-stage nuclei images obtained with antibodies against some of these proteins, such as RAD51 and RPA2 (Cole et al., 2012; Gil-Fernandez et al., 2021), can benefit from the use of our macro as shown in Figure 4 and Supplementary Figure S1; these foci appear at earlier stages and significant presence at pachytene stage reflects a problem in DSB repair. In addition, the application also successfully analyzes images obtained with specific centromere markers (e.g., CREST serum), which are often employed in meiosis studies (Segura et al., 2013) (Figure 4 and Supplementary Figure S1). Centromere identification is not a requirement to obtain SC and CO data, but whether they are identified by DAPI or by CREST serum, centromeres can be used as SC measurement reference points.

In summary, Synaptonemal & CO analyzer is a versatile tool for recombination studies in vertebrate nuclei immunostained with diverse antibodies: it can be used in experiments analyzing SCs only, or SCs plus COs, and it will work with various stainings and antibodies. Unlike other applications (Peterson et al., 2019), it provides means to discriminate overlapping SCs and to exclude sex chromosomes from the analysis without further data loss. In addition, results can be easily verified: the software creates a results folder with an image, a table and a set of files. The results image contains the analyzed structures and merges all analyzed channels. The results table provides all relevant recombination meiotic studies’ data. Finally, there is a file for all detected structures that allows for overlaying them to the results image, enabling visual inspection and verification (Figure 3).

### Other advantages of the application

The macro has several additional advantages: 1) it is free, has been released under an open-source license (GNU General Public License), is accessible through stable public repositories (https://github.com/joaquim-soriano/Synaptonemal-and-CO-analyzer, https://zenodo.org) and has been assigned a DOI (https://zenodo.org/badge/latestdoi/410606632). 2) It is very intuitive and the learning process is facilitated by a user manual and video tutorials provided as Supplementary Material and at https://github.com/joaquim-soriano/Synaptonemal-and-CO-analyzer. Further support about ImageJ/FIJI can be received by using the wikis (ImageJ Information and Documentation Portal, n.d.; FIJI Software, n.d.) and mailing lists (ImageJ, n.d.; FIJI, n.d.) indicated in the bibliography. 3) It has been developed under ImageJ/FIJI (running on Java), which is free, open-source, well documented and ensures operative system compatibility (Windows and MacOS). It is also the most popular image analysis and processing software in biological science (Cardona and Tomancak, 2012; Eliceiri et al., 2012; Schindelin et al., 2012; Schneider et al., 2012). By using ImageJ scripting language, Synaptonemal & CO Analyzer can reach a large number of users that might get involved in further software’s development.

## Conclusion

Our application will facilitate studies about the genetic, epigenetic and environmental factors that affect the recombination rate and, hence, that can increase the frequency of chromosomal abnormalities and fertility problems. Among the environmental effects that affect recombination in mice, bisphenol A (an endocrine disruptor found in plastics used in a wide variety of consumer products) has been an object of study for a long time (Hunt et al., 2003; Susiarjo et al., 2007; Vrooman et al., 2015). These findings motivated us to search and identify a new effector, diet, in a study that was substantially accelerated by our application (Belmonte-Tebar et al., 2022). We continue successfully using it in our current research about recombination in male mice (Belmonte-Tebar et al., in preparation), proving that Synaptonemal & CO Analyzer performs very well, not only in a theoretical, controlled environment, but also with real complex data.

Synaptonemal & CO Analyzer meets an important need in the recombination field by providing an efficient and consistent tool for the analysis of SC length and COs number and distribution. Unlike other applications, it is free, hosted on an archivally stable platform, well documented and intuitive, runs in most computers and does not require computational skills or extensive training, thus facilitating usability (Carpenter et al., 2012), rigor and reproducibility of the analyses (Brito et al., 2020).

More importantly, the application facilitates the analysis of pachytene nuclei from diverse vertebrate species immunostained with different antibodies and centromere identification methods. In summary, Synaptonemal & CO Analyzer is a novel and versatile application tool for the study of recombination that is accessible for future improvements.

## Data Availability

The raw data supporting the conclusion of this article will be made available by the authors, without undue reservation.
