# Correction: Synaptonemal & CO analyzer: a tool for synaptonemal complex and crossover analysis in immunofluorescence images

**DOI:** 10.3389/fcell.2025.1736251

**Published:** 2025-12-10

**Authors:** Joaquim Soriano, Angela Belmonte-Tebar, Elena de la Casa-Esperon

**Affiliations:** 1 Centro Regional de Investigaciones Biomédicas (CRIB), Universidad de Castilla-La Mancha, Albacete, Spain; 2 Biology of Cell Growth, Differentiation and Activation Group, Department of Inorganic and Organic Chemistry and Biochemistry, School of Pharmacy, Universidad de Castilla-La Mancha and IDISCAM, Albacete, Spain

**Keywords:** meiotic recombination, crossover, synaptonemal complex, image analysis, imagej, Fiji, open-source software

**Affiliation “2** Biology of Cell Growth, Differentiation and Activation Group, Department of Inorganic and Organic Chemistry and Biochemistry, School of Pharmacy, Universidad de Castilla-La Mancha and IDISCAM, Albacete, Spain” was erroneously given as “Biology of Cell Growth, Differentiation and Activation Group, Department of Inorganic and Organic Chemistry and Biochemistry, School of Pharmacy, Universidad de Castilla-La Mancha, Albacete, Spain”.

The original article has been updated.

