# Supplementary material for: Synaptonemal & CO analyzer: A tool for synaptonemal complex and crossover analysis in immunofluorescence images
Source: Front Cell Dev Biol. 2023 Jan 19;11:1005145. doi: 10.3389/fcell.2023.1005145 (PMC9894712; doi:10.3389/fcell.2023.1005145)
Supplement: Supplementary file 1 [file DataSheet2.PDF]

Supplementary Figures:

- Supplementary Figure 1: Synaptonemal & CO Analyzer is a versatile tool for the analysis of crossover number and distribution, and synaptonemal complex length in immunostained pachytene cells.
- Supplementary Figure 2: Synaptonemal & CO Analyzer’s process and validation results. Flow diagram of the general process.
- Supplementary Figure 3: Synaptonemal & CO Analyzer’s process and validation results. Flow diagram of the semiautomatic process.

Supplementary Figure 1: Synaptonemal & CO Analyzer is a versatile tool for the analysis of crossover number and distribution, and synaptonemal complex length in immunostained pachytene cells.

Images from pachytene-stage nuclei were successfully analyzed with the macro, demonstrating it performs well with diverse vertebrate species, fluorochromes, central or distal centromeres stained with DAPI or CREST serum, and different antibodies for CO and SC identification. In all cases, the software allows the semiautomatic identification of SCs (white lines), COs (yellow circles) or centromeres (white circles), and calculates the SC length and the CO number and distribution along the SC starting from one SC end (arrow heads) and to the centromere. For each analyzed image, the following information is provided: species, labels and colors, authors and a zoomed view showing the elements detected by the macro, as well as a table with the results of the analysis. The first column of the table identifies each SC in the image. The following columns show the total length of each SC and the total number of COs per SC (the sum of the SC lengths and the total number of COs per nucleus are shown at the bottom). Partial lengths represent SC distances starting from one SC end to the first CO, from this to the next CO (if applicable) and so on, and from the last CO to the opposite SC end. The starting point of each SC measurement (arrow heads) is the upper end of that SC by default. In telocentric organisms (such as most house mice), DAPI labeling allows to identify the centromeric end of each SC, which becomes the starting point for SC length measures. When CREST serum is used to locate centromeres position, the macro provides additional data for each of the two chromosome arms. In this case, partial length measurements start from the upper end of the SC up to the centromere (arm 1, in negative values), from which arm 2 measurements (positive values) begin up to the other extreme. When necessary, SC and foci signal were detected using the skeletonize SC macro recorder and the foci detection macro recorder (see Supplementary Materials, User Manual). RPA and RAD51 signal detection relied on the latter, using the Find maxima analysis option. SC lengths are shown in µm when donated image files contain the metadata (e.g., images A and G), in pixels when lost.

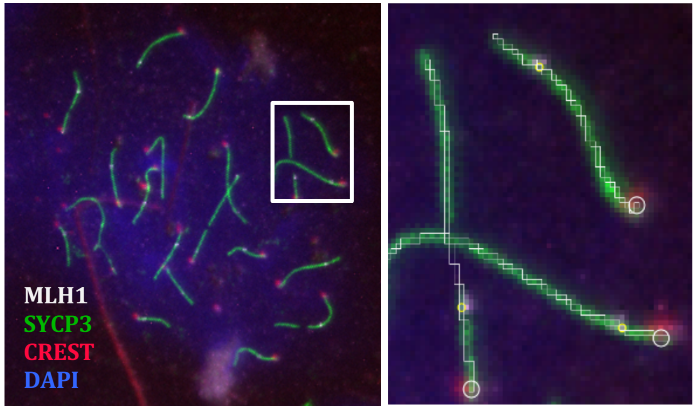

---SC---	Total length	COs number	Partial length-1	Partial length-2	Centromere position	CO number arm 1	CO number arm 2	arm1: partial length1	arm1: partial length2	arm2: partial length1	arm2: partial length2
SC-1	6.753	1	6.000	0.753	0.089	0	1	-0.089	0.000	5.91	0.753
SC-2	7.246	1	6.296	0.949	0.089	0	1	-0.089	0.000	6.207	0.949
SC-3	6.966	1	2.119	4.847	6.776	1	0	-2.119	-4.657	0.19	0
SC-4	6.426	1	1.543	4.882	6.336	1	0	-1.543	-4.793	0.089	0
SC-5	8.450	0	8.450	0.000	0.395	0	0	-0.395	0.000	8.055	0
SC-6	6.626	1	2.145	4.480	6.130	1	0	-2.145	-3.985	0.495	0
SC-7	7.171	1	4.917	2.255	0.089	0	1	-0.089	0.000	4.827	2.255
SC-8	4.190	1	1.059	3.132	0.089	0	1	-0.089	0.000	0.969	3.131
SC-9	6.041	1	1.718	4.324	0.063	0	1	-0.063	0.000	1.654	4.324
SC-10	4.832	1	2.228	2.604	4.210	1	0	-2.228	-1.982	0.622	0
SC-11	7.656	1	1.565	6.091	7.593	1	0	-1.565	-6.028	0.063	0
SC-12	4.671	0	4.671	0.000	0.369	0	0	-0.369	0.000	4.302	0
SC-13	9.192	1	3.856	5.336	0.063	0	1	-0.063	0.000	3.793	5.335
SC-14	3.758	1	0.749	3.009	0.063	0	1	-0.063	0.000	0.685	3.009
SC-15	6.176	1	1.813	4.363	0.190	0	1	-0.190	0.000	1.624	4.363
SC-16	10.978	1	0.190	10.788	10.788	1	0	-0.190	-10.598	0.19	0
SC-17	9.127	0	9.127	0.000	0.089	0	0	-0.089	0.000	9.037	0
SC-18	10.275	1	8.968	1.307	10.085	1	0	-8.968	-1.117	0.19	0
SC-19	8.698	1	6.505	2.193	8.635	1	0	-6.505	-2.130	0.063	0
SUM	135.231	16									

A) Wild-captured house mouse (*Mus musculus domesticus)* with a standard karyotype (courtesy of Cristina Marín and Aurora Ruiz-Herrera, Universitat Autonoma de Barcelona, Spain (Vara et al. 2021)).

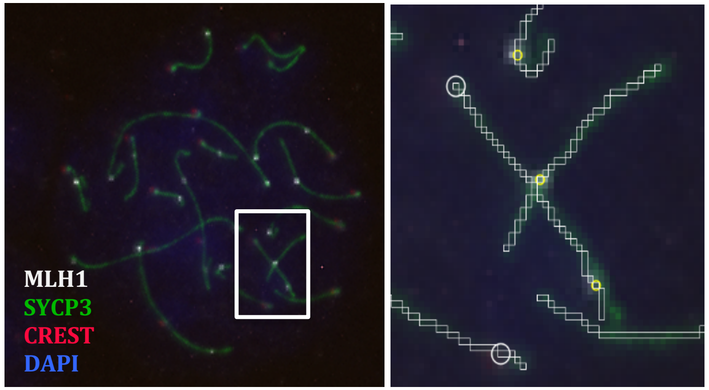

---SC---	Total length	COs number	Partial length-1	Partial length-2	Partial length-3	Centromere position	CO number arm 1	CO number arm 2	arm1: partial length1	arm1: partial length2	arm1: partial length3	arm2: partial length1	arm2: partial length2	arm2: partial length3
SC-1	31.10	1	1.82	29.28	0.00	30.66	1	0	-1.82	-28.84	0	0.44	0.00	0
SC-2	34.96	0	34.96	0.00	0.00	0.44	0	0	-0.44	0.00	0	34.52	0.00	0
SC-3	18.11	0	18.11	0.00	0.00	17.66	0	0	-17.67	0.00	0	0.44	0.00	0
SC-4	116.38	2	9.13	106.93	0.31	67.58	1	1	-9.13	-58.45	0	48.48	0.31	0
SC-5	52.42	2	15.83	36.28	0.31	0.44	0	2	-0.44	0.00	0	15.39	36.28	0.31
SC-6	29.23	0	29.23	0.00	0.00	0.94	0	0	-0.94	0.00	0	28.29	0.00	0
SC-7	52.98	1	21.18	31.80	0.00	51.42	1	0	-21.18	-30.24	0	1.56	0.00	0
SC-8	18.08	1	10.38	7.71	0.00	0.44	0	1	-0.44	0.00	0	9.94	7.71	0
SC-9	36.58	1	7.62	28.96	0.00	0.44	0	1	-0.44	0.00	0	7.18	28.96	0
SC-10	15.69	1	13.25	2.45	0.00	0.44	0	1	-0.44	0.00	0	12.80	2.45	0
SC-11	52.27	1	45.04	7.23	0.00	3.72	0	1	-3.72	0.00	0	41.32	7.23	0
SC-12	19.62	0	19.62	0.00	0.00	16.16	0	0	-16.16	0.00	0	3.46	0.00	0
SC-13	32.48	0	32.48	0.00	0.00	30.04	0	0	-30.04	0.00	0	2.45	0.00	0
SC-14	77.75	2	4.58	63.95	9.21	40.12	1	1	-4.58	-35.54	0	28.41	9.21	0
SC-15	89.88	1	2.19	87.69	0.00	49.70	1	0	-2.19	-47.51	0	40.19	0.00	0
SC-16	28.20	1	19.33	8.87	0.00	0.00	0	0	0.00	0.00	0	0.00	0.00	0
SC-17	30.61	1	26.91	3.70	0.00	0.44	0	1	-0.44	0.00	0	26.47	3.70	0
SUM	736.34	15												

B) Wild-captured house mouse (*Mus musculus domesticus)* with Robertsonian translocations (courtesy of Cristina Marin and Aurora Ruiz-Herrera, Universitat Autonoma de Barcelona, Spain (Vara et al. 2021).

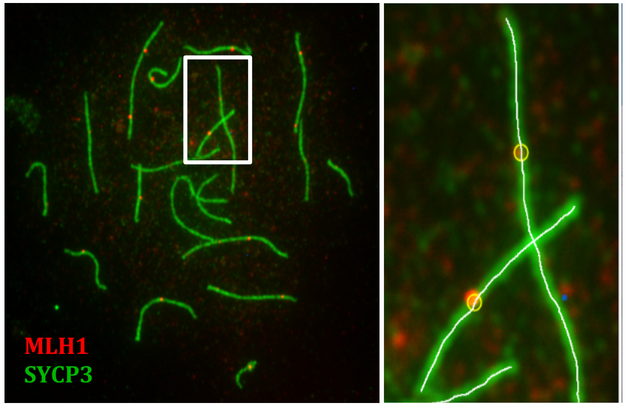

---SC---	Total length	COs number	Partial length-1	Partial length-2	Partial length-3
SC-1	620.01	2	165.47	352.07	102.47
SC-2	505.63	2	103.61	371.07	30.95
SC-3	443.08	1	292.28	150.80	0
SC-4	392.27	1	255.58	136.69	0
SC-5	496.79	1	188.23	308.55	0
SC-6	399.72	1	208.24	191.48	0
SC-7	224.25	1	54.31	169.94	0
SC-8	359.42	1	311.43	47.99	0
SC-9	648.97	2	248.74	169.04	231.19
SC-10	353.97	1	102.78	251.19	0
SC-11	508.86	2	56.92	379.23	72.71
SC-12	501.58	1	227.32	274.26	0
SC-13	228.91	1	5.54	223.37	0
SC-14	266.15	1	150.99	115.16	0
SC-15	531.50	1	211.04	320.46	0
SC-16	644.63	2	160.78	369.97	113.88
SC-17	313.62	1	181.37	132.25	0
SUM	7439.35	22			

C) Matthey's mouse (*Mus matheyi*, courtesy of Jesus Page (Universidad Autonoma de Madrid, Spain) and Frederic Veyrunes (Universite Montpellier, France).

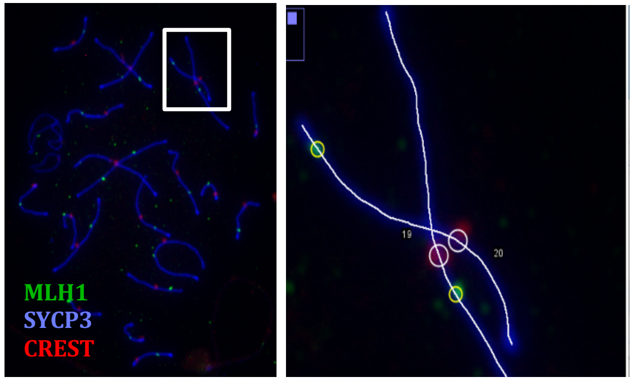

---SC---	Total length	COs number	Partial length-1	Partial length-2	Partial length-3	Centromere position	CO number arm 1	CO number arm 2	arm1: partial length1	arm1: partial length2	arm1: partial length3	arm2: partial length1	arm2: partial length2
SC-1	379.05	1	90.3345	288.7142	0	216.77	1	0	-90.34	-126.44	0	162.28	0.00
SC-2	299.54	1	231.58	67.96	0	274.48	1	0	-231.58	-42.90	0	25.06	0.00
SC-3	516.48	1	345.02	171.45	0	139.04	0	1	-139.04	0.00	0	205.98	171.45
SC-4	542.48	1	385.26	157.22	0	220.15	0	1	-220.15	0.00	0	165.11	157.22
SC-5	212.10	1	89.69	122.41	0	181.12	1	0	-89.69	-91.43	0	30.99	0.00
SC-6	419.85	1	189.40	230.45	0	2.12	0	1	-2.12	0.00	0	187.28	230.45
SC-7	632.40	1	201.11	431.29	0	385.77	1	0	-201.11	-184.66	0	246.63	0.00
SC-8	343.01	1	295.44	47.57	0	103.86	0	1	-103.86	0.00	0	191.58	47.57
SC-9	361.45	1	82.78	278.67	0	134.34	1	0	-82.78	-51.56	0	227.11	0.00
SC-10	928.99	1	521.23	407.76	0	396.45	0	1	-396.45	0.00	0	124.78	407.76
SC-11	452.43	1	362.20	90.23	0	251.99	0	1	-251.99	0.00	0	110.21	90.23
SC-12	291.76	1	18.61	273.15	0	119.42	1	0	-18.61	-100.81	0	172.34	0.00
SC-13	241.97	1	138.70	103.27	0	241.47	1	0	-138.70	-102.77	0	0.50	0.00
SC-14	323.17	1	111.06	212.12	0	284.52	1	0	-111.06	-173.47	0	38.65	0.00
SC-15	545.09	1	444.67	100.42	0	342.75	0	1	-342.75	0.00	0	101.92	100.42
SC-16	579.87	1	386.11	193.77	0	0.00	0	0	0.00	0.00	0	0.00	0.00
SC-17	449.56	1	38.82	410.74	0	287.31	1	0	-38.82	-248.49	0	162.25	0.00
SC-18	735.40	1	384.51	350.89	0	327.57	0	1	-327.57	0.00	0	56.94	350.89
SC-19	762.10	0	762.10	0.00	0	372.11	0	0	-372.11	0.00	0	389.99	0.00
SC-20	816.80	2	272.88	307.79	236.13	0.00	0	0	0.00	0.00	0	0.00	0.00
SC-21	580.78	1	455.64	125.14	0	308.13	0	1	-308.13	0.00	0	147.51	125.14
SUM	10414.27	21											

D) Mongolian gerbil (*Meriones unguiculatus,* courtesy of Jesus Page, Universidad Autonoma de Madrid, Spain).

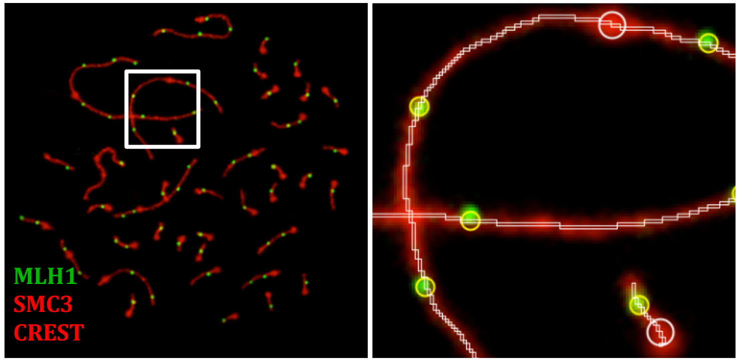

---SC---	Total length	COs number	Partial length-1	Partial length-2	Partial length-3	Partial length-4	Partial length-5	Partial length-6	Partial length-7	Partial length-8	Centromere position	CO number arm 1	CO number arm 2	arm1: partial length1	arm1: partial length2	arm1: partial length3	arm1: partial length4	arm1: partial length5	arm2: partial length1	arm2: partial length2	arm2: partial length3	arm2: partial length4	arm2: partial length5
SC-1	2,301,604	4	177,426	624,558	619,706	693,848	186,066	0	0	0	69,142	0	4	-6,914	0	0	0	0	10,828	62,456	61,971	69,385	18,607
SC-2	295,563	1	31,213	26,435	0	0	0	0	0	0	27,435	1	0	-3,121	-24,314	0	0	0	2,121	0	0	0	0
SC-3	268,995	1	65,355	20,364	0	0	0	0	0	0	23,364	1	0	-6,535	-16,829	0	0	0	3,535	0	0	0	0
SC-4	32,799	1	141,569	186,421	0	0	0	0	0	0	298,848	1	0	-14,157	-15,728	0	0	0	2,914	0	0	0	0
SC-5	357,279	1	217,426	139,853	0	0	0	0	0	0	283,995	1	0	-21,743	-6,657	0	0	0	7,328	0	0	0	0
SC-6	328,345	1	149,853	178,492	0	0	0	0	0	0	2.5	0	1	-2,500	0	0	0	0	12,485	17,849	0	0	0
SC-7	404,914	1	315,772	89,142	0	0	0	0	0	0	35,355	0	1	-3,535	0	0	0	0	28,042	8,914	0	0	0
SC-8	653,198	2	116,066	233,137	303,995	0	0	0	0	0	21,213	0	2	-2,121	0	0	0	0	9,485	23,314	30,399	0	0
SC-9	257,635	1	83,284	17,435	0	0	0	0	0	0	202,279	1	0	-8,328	-11,900	0	0	0	5,535	0	0	0	0
SC-10	388,995	1	217,426	171,569	0	0	0	0	0	0	383,995	1	0	-21,743	-16,657	0	0	0	0.5	0	0	0	0
SC-11	1,646,812	1	43,284	1,603,528	0	0	0	0	0	0	735,122	1	0	-4,328	-69,184	0	0	0	91,169	0	0	0	0
SC-12	907,756	2	276,421	323,848	307,487	0	0	0	0	0	169,853	0	2	-16,985	0	0	0	0	10,657	32,385	30,749	0	0
SC-13	609,767	1	489,914	119,853	0	0	0	0	0	0	6,364	0	1	-6,364	0	0	0	0	42,627	11,985	0	0	0
SC-14	265,563	1	0.5	260,563	0	0	0	0	0	0	260,563	1	0	-0.5	-25,556	0	0	0	0.5	0	0	0	0
SC-15	323,848	1	57,426	266,421	0	0	0	0	0	0	280,563	1	0	-5,743	-22,314	0	0	0	4,328	0	0	0	0
SC-16	48,935	1	147,782	341,569	0	0	0	0	0	0	0.7071	0	1	-0.707	0	0	0	0	14,071	34,157	0	0	0
SC-17	32,935	1	141,924	187,426	0	0	0	0	0	0	300,208	1	0	-14,192	-15,828	0	0	0	2,914	0	0	0	0
SC-18	510,061	1	133,284	376,777	0	0	0	0	0	0	39,142	0	1	-3,914	0	0	0	0	9,414	37,678	0	0	0
SC-19	532,132	1	309,853	222,279	0	0	0	0	0	0	507,132	1	0	-30,985	-19,728	0	0	0	2,500	0	0	0	0
SC-20	460,061	1	151,569	308,492	0	0	0	0	0	0	43,284	0	1	-4,328	0	0	0	0	10,829	30,849	0	0	0
SC-21	568,345	2	0.5	256,569	306,777	0	0	0	0	0	430,563	2	0	-0.5	-25,657	-16,899	0	0	13,778	0	0	0	0
SC-22	257,279	1	63,284	193,995	0	0	0	0	0	0	252,279	1	0	-6,328	-18,900	0	0	0	0.5	0	0	0	0
SC-23	80,533	2	223,995	326,274	255,061	0	0	0	0	0	0.5	0	2	-0.5	0	0	0	0	21,899	32,627	25,506	0	0
SC-24	261,066	1	73,284	187,782	0	0	0	0	0	0	253,995	1	0	-7,328	-18,071	0	0	0	0.707	0	0	0	0
SC-25	44,799	2	77,426	221,421	149,142	0	0	0	0	0	418,848	2	0	-7,743	-22,142	-12,000	0	0	2,914	0	0	0	0
SC-26	214,853	1	119,497	95,355	0	0	0	0	0	0	55,355	0	1	-5,535	0	0	0	0	6,414	9,536	0	0	0
SC-27	841,899	2	159,853	507,696	17,435	0	0	0	0	0	29,299	1	1	-15,985	-13,314	0	0	0	37,456	17,435	0	0	0
SC-28	295,563	1	131,924	16,364	0	0	0	0	0	0	250,208	1	0	-13,192	-11,828	0	0	0	4,536	0	0	0	0
SC-29	56,734	1	189,853	377,487	0	0	0	0	0	0	0.5	0	1	-0.5	0	0	0	0	18,485	37,749	0	0	0
SC-30	144,196	2	688,553	61,598	137,426	0	0	0	0	0	115,711	0	2	-11,571	0	0	0	0	57,284	61,598	13,743	0	0
SC-31	764,264	2	290,208	281,421	192,635	0	0	0	0	0	12,364	0	2	-12,364	0	0	0	0	16,657	28,142	19,264	0	0
SC-32	344,497	1	163,284	181,213	0	0	0	0	0	0	313,284	1	0	-16,328	-15,000	0	0	0	3,121	0	0	0	0
SC-33	439,706	1	157,426	282,279	0	0	0	0	0	0	376,421	1	0	-15,743	-21,899	0	0	0	6,328	0	0	0	0
SC-34	316,924	1	241,569	75,355	0	0	0	0	0	0	0.5	0	1	-0.5	0	0	0	0	23,657	7,535	0	0	0
SC-35	248,995	1	243,995	0.5	0	0	0	0	0	0	0.5	0	1	-0.5	0	0	0	0	23,899	0.5	0	0	0
SC-36	3,222,559	4	18,435	648,406	1,144,975	649,706	595,122	0	0	0	1,177,609	2	2	-18,435	-64,841	-34,485	0	0	80,012	64,971	59,512	0	0
SC-37	4,142,498	7	3.5	383,137	579,706	911,543	565,269	582,426	953,848	131,569	171,968	3	4	-3,500	-38,314	-57,971	-72,184	0	18,971	56,527	58,243	95,385	13,157
SC-38	1,901,016	4	15,435	471,127	927,401	214,853	133,284	0	0	0	1,430,452	2	2	-15,435	-47,113	-80,498	0	0	12,243	21,485	13,328	0	0
SC-39	363,848	1	211,924	151,924	0	0	0	0	0	0	0.7071	0	1	-0.707	0	0	0	0	20,485	15,192	0	0	0
SUM	2,910,515	62																					

E) Chicken (*Gallus gallus*, courtesy of María Inés Pigozzi, Instituto de Investigaciones Biomédicas, Universidad de Buenos Aires-CONICET, Argentina (del Priore and Pigozzi, 2020)). Centromeres appear as red bulging dots and were automatically detected by the macro. In order to tell apart SCs and centromeres, the red channel was duplicated in the blue channel. The original red channel was used for SC detection and the blue one for centromere detection using the Find maxima algorithm built in imageJ.

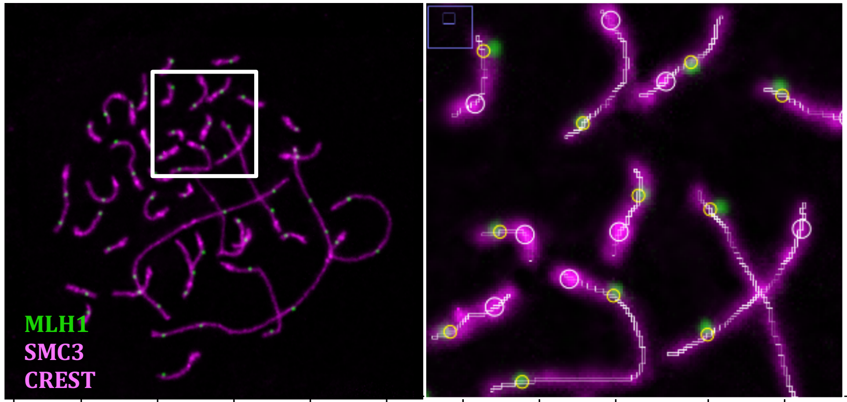

F) Duck (*Anas platyrhynchos*, courtesy of María Inés Pigozzi, Instituto de Investigaciones Biomédicas, Universidad de Buenos Aires-CONICET, Argentina (Pigozzi and del Priore, 2016)). Centromeres are seen as dots bulging from the SCs. SCs and centromeres were analyzed as described above in Figure E legend.

---SC---	Total length	COs number	Partial length-1	Partial length-2	Partial length-3	Partial length-4	Partial length-5	Centromere position	CO number arm 1	CO number arm 2	arm1: partial length1	arm1: partial length2	arm1: partial length3	arm1: partial length4	arm1: partial length5	arm2: partial length1	arm2: partial length2	arm2: partial length3	arm2: partial length4	arm2: partial length5
SC-1	2.964	1	2.102	0.862	0	0	0	0.570	0	1	-0.570	0.000	0	0	0	1.531	0.862	0	0	0
SC-2	4.170	1	1.793	2.377	0	0	0	3.168	1	0	-1.793	-1.375	0	0	0	1.002	0.000	0	0	0
SC-3	4.913	1	0.894	4.019	0	0	0	4.328	1	0	-0.894	-3.434	0	0	0	0.585	0.000	0	0	0
SC-4	2.477	1	1.219	1.258	0	0	0	0.614	0	1	-0.614	0.000	0	0	0	0.605	1.258	0	0	0
SC-5	6.933	2	1.444	4.066	1.424	0	0	6.267	2	0	-1.444	-4.066	-0.757	0	0	0.667	0.000	0	0	0
SC-6	2.431	1	0.274	2.157	0	0	0	1.846	1	0	-0.274	-1.572	0	0	0	0.585	0.000	0	0	0
SC-7	2.320	1	1.289	1.031	0	0	0	0.486	0	1	-0.486	0.000	0	0	0	0.803	1.031	0	0	0
SC-8	5.265	2	0.035	3.384	1.846	0	0	4.711	2	0	-0.035	-3.384	-1.293	0	0	0.553	0.000	0	0	0
SC-9	3.634	1	0.582	3.052	0	0	0	3.221	1	0	-0.582	-2.638	0	0	0	0.414	0.000	0	0	0
SC-10	3.198	1	3.093	0.105	0	0	0	0.317	0	1	-0.317	0.000	0	0	0	2.776	0.105	0	0	0
SC-11	7.491	2	3.395	3.470	0.626	0	0	0.344	0	2	-0.344	0.000	0	0	0	3.051	3.470	0.626	0	0
SC-12	4.843	1	0.667	4.176	0	0	0	3.812	1	0	-0.667	-3.145	0	0	0	1.031	0.000	0	0	0
SC-13	4.151	1	1.248	2.903	0	0	0	3.467	1	0	-1.248	-2.219	0	0	0	0.684	0.000	0	0	0
SC-14	2.103	1	1.998	0.105	0	0	0	0.681	0	1	-0.681	0.000	0	0	0	1.317	0.105	0	0	0
SC-15	12.925	1	12.538	0.387	0	0	0	3.532	0	1	-3.532	0.000	0	0	0	9.006	0.387	0	0	0
SC-16	2.861	1	0.768	2.092	0	0	0	2.206	1	0	-0.768	-1.438	0	0	0	0.655	0.000	0	0	0
SC-17	5.462	1	5.020	0.443	0	0	0	0.105	0	1	-0.105	0.000	0	0	0	4.915	0.443	0	0	0
SC-18	2.619	1	0.373	2.247	0	0	0	1.740	1	0	-0.373	-1.368	0	0	0	0.879	0.000	0	0	0
SC-19	2.434	1	0.148	2.285	0	0	0	1.750	1	0	-0.148	-1.601	0	0	0	0.684	0.000	0	0	0
SC-20	3.031	1	1.692	1.340	0	0	0	0.935	0	1	-0.934	0.000	0	0	0	0.757	1.340	0	0	0
SC-21	3.217	1	1.680	1.537	0	0	0	2.256	1	0	-1.680	-0.576	0	0	0	0.961	0.000	0	0	0
SC-22	5.009	1	4.523	0.486	0	0	0	0.768	0	1	-0.768	0.000	0	0	0	3.755	0.486	0	0	0
SC-23	6.022	1	0.274	5.749	0	0	0	5.240	1	0	-0.274	-4.966	0	0	0	0.783	0.000	0	0	0
SC-24	2.079	1	0.652	1.427	0	0	0	1.258	1	0	-0.652	-0.605	0	0	0	0.821	0.000	0	0	0
SC-25	2.318	1	2.143	0.175	0	0	0	0.553	0	1	-0.553	0.000	0	0	0	1.589	0.175	0	0	0
SC-26	5.164	1	2.302	2.862	0	0	0	0.346	0	1	-0.346	0.000	0	0	0	1.956	2.862	0	0	0
SC-27	3.051	1	2.763	0.288	0	0	0	1.033	0	1	-1.033	0.000	0	0	0	1.729	0.288	0	0	0
SC-28	9.907	2	3.006	6.389	0.513	0	0	0.000	0	0	0.000	0.000	0	0	0	0.000	0.000	0	0	0
SC-29	1.741	1	0.315	1.427	0	0	0	1.060	1	0	-0.315	-0.745	0	0	0	0.681	0.000	0	0	0
SC-30	2.556	1	1.019	1.537	0	0	0	1.735	1	0	-1.019	-0.716	0	0	0	0.821	0.000	0	0	0
SC-31	5.716	1	5.302	0.414	0	0	0	0.911	0	1	-0.910	0.000	0	0	0	4.392	0.414	0	0	0
SC-32	4.946	1	4.463	0.484	0	0	0	0.923	0	1	-0.922	0.000	0	0	0	3.540	0.483	0	0	0
SC-33	4.119	1	3.872	0.247	0	0	0	0.862	0	1	-0.862	0.000	0	0	0	3.010	0.247	0	0	0
SC-34	33.738	4	0.920	10.515	12.696	8.897	0.710	21.167	2	2	-0.920	-10.515	-9.732	0	0	2.964	8.897	0.710	0	0
SC-35	21.532	4	3.284	10.038	2.720	4.807	0.684	0.780	0	4	-0.780	0.000	0	0	0	2.503	10.038	2.720	4.807	0.684
SC-36	12.842	3	0.696	5.706	5.195	1.246	0	12.231	3	0	-0.696	-5.706	-5.195	-0.634	0	0.611	0.000	0	0	0
SC-37	3.287	0	3.287	0.000	0	0	0	2.871	0	0	-2.871	0.000	0	0	0	0.416	0.000	0	0	0
SC-38	11.836	2	6.421	5.072	0.344	0	0	0.836	0	2	-0.836	0.000	0	0	0	5.585	5.072	0.344	0	0
SC-39	3.058	1	1.950	1.108	0	0	0	0.710	0	1	-0.710	0.000	0	0	0	1.240	1.108	0	0	0
SC-40	16.535	2	2.555	5.687	8.293	0	0	16.290	2	0	-2.555	-5.687	-8.048	0	0	0.245	0.000	0	0	0
SC-41	7.240	1	5.406	1.834	0	0	0	0.315	0	1	-0.315	0.000	0	0	0	5.091	1.834	0	0	0
SUM	252.139	54																		

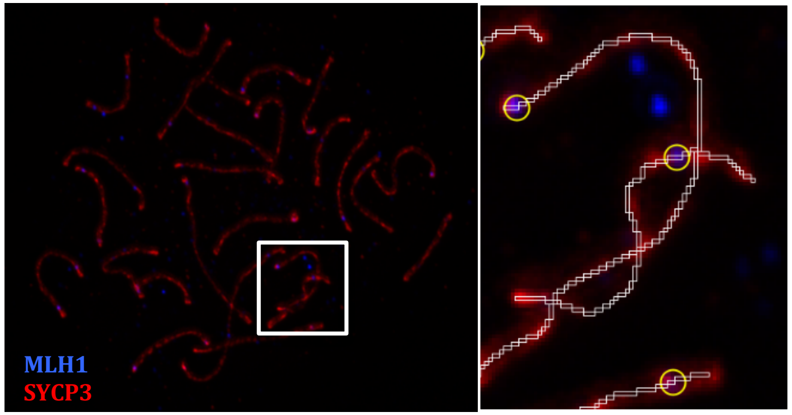

---SC---	Total length	COs number	Partial length-1	Partial length-2	Partial length-3	Partial length-4	Partial length-5
SC-1	7.2758	1	6.9841	0.2917	0	0	0
SC-2	7.4565	3	2.0065	3.8658	0.8206	0.7636	0
SC-3	6.9851	1	6.8878	0.09725	0	0	0
SC-4	6.8934	2	1.298	5.3282	0.2672	0	0
SC-5	6.7322	1	3.2545	3.4777	0	0	0
SC-6	8.9929	1	8.0728	0.9201	0	0	0
SC-7	9.9589	1	9.9265	0.03242	0	0	0
SC-8	8.9531	1	7.436	1.517	0	0	0
SC-9	8.2936	2	0.04584	1.241	7.0067	0	0
SC-10	7.4834	2	0.6048	6.4952	0.3834	0	0
SC-11	6.5048	0	6.5048	0	0	0	0
SC-12	7.971	1	0.3455	7.6255	0	0	0
SC-13	9.2365	4	2.6863	1.0688	4.4696	0.6909	0.3209
SC-14	9.6551	2	0.6071	8.5326	0.5154	0	0
SC-15	4.8855	0	4.8855	0	0	0	0
SC-16	7.7366	2	0.04584	7.6583	0.03242	0	0
SC-17	15.004	2	8.5386	4.5659	1.8995	0	0
SC-18	10.4154	1	9.2582	1.1572	0	0	0
SC-19	9.4732	1	0.04584	9.4274	0	0	0
SC-20	8.8805	1	0.4617	8.4188	0	0	0
SC-21	6.8374	1	1.611	5.2263	0	0	0
SC-22	11.732	1	0.2269	11.5051	0	0	0
SC-23	13.5186	1	0.4506	13.068	0	0	0
SC-24	16.0451	3	1.4545	7.989	5.8783	0.7233	0
SC-25	10.743	1	0.6696	10.0734	0	0	0
SUM	227.664	36	----	----	----	----	----

G) Zebrafish (*Danio rerio*, courtesy of Yukiko Imai, National Institute of Genetics, Japan).

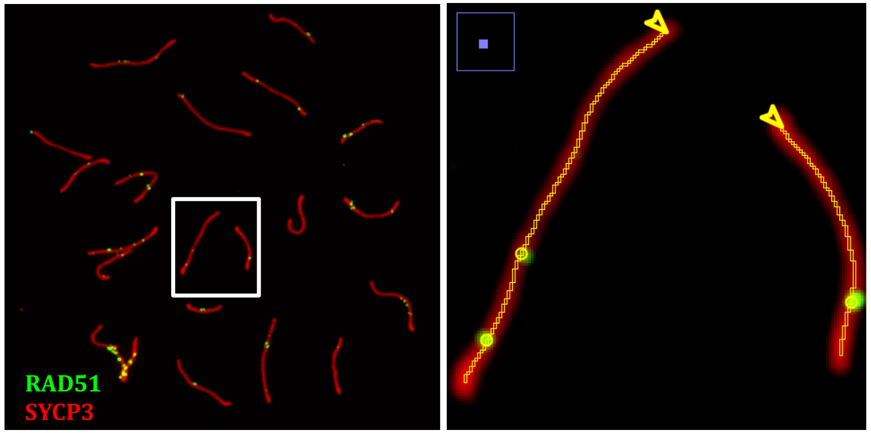

---SC---	Total length	COs number	Partial length-1	Partial length-2	Partial length-3	Partial length-4	Partial length-5	Partial length-6	Partial length-7	Partial length-8	Partial length-9
SC-1	119.20	2	97.31	5.24	16.64	0.00	0.00	0	0	0	0
SC-2	205.44	6	47.10	7.07	34.38	30.66	9.00	13.00	64.23	0	0
SC-3	172.00	4	11.19	24.56	13.49	106.54	16.23	0	0	0	0
SC-4	190.17	1	8.78	181.39	0.00	0.00	0.00	0	0	0	0
SC-5	101.23	6	5.12	6.00	6.41	21.73	35.46	16.31	10.19	0	0
SC-6	141.78	1	0.50	141.28	0.00	0.00	0.00	0	0	0	0
SC-7	135.89	2	47.47	36.28	52.13	0.00	0.00	0	0	0	0
SC-8	172.13	5	35.30	27.90	62.46	5.24	24.80	16.435	0	0	0
SC-9	130.50	0	130.50	0.00	0.00	0.00	0.00	0	0	0	0
SC-10	145.82	4	16.64	6.66	4.24	94.60	23.68	0	0	0	0
SC-11	163.41	2	107.21	37.38	18.81	0.00	0.00	0	0	0	0
SC-12	98.25	1	77.10	21.16	0.00	0.00	0.00	0	0	0	0
SC-13	166.50	7	17.44	20.56	24.56	23.31	10.24	26.24	36.24	7.91	0
SC-14	119.92	3	21.44	9.66	81.91	6.91	0.00	0	0	0	0
SC-15	167.47	6	85.06	10.49	12.49	20.66	4.41	19.00	15.36	0	0
SC-16	76.49	3	32.26	6.00	10.83	27.40	0.00	0	0	0	0
SC-17	2.00	0	2.00	0.00	0.00	0.00	0.00	0	0	0	0
SC-18	193.95	4	38.26	18.66	8.83	102.80	25.40	0	0	0	0
SC-19	154.30	8	76.13	4.83	9.66	9.66	4.83	7.66	15.73	19.90	5.91
SC-20	132.19	6	39.19	18.90	18.14	16.31	8.83	20.90	9.91	0	0
SC-21	127.81	1	17.78	110.03	0.00	0.00	0.00	0	0	0	0
SC-22	162.44	2	68.55	4.83	89.07	0.00	0.00	0	0	0	0
SUM	3078.88	74									

H) House mouse (*Mus musculus*, courtesy of Jesus Page, Universidad Autonoma de Madrid, Spain)

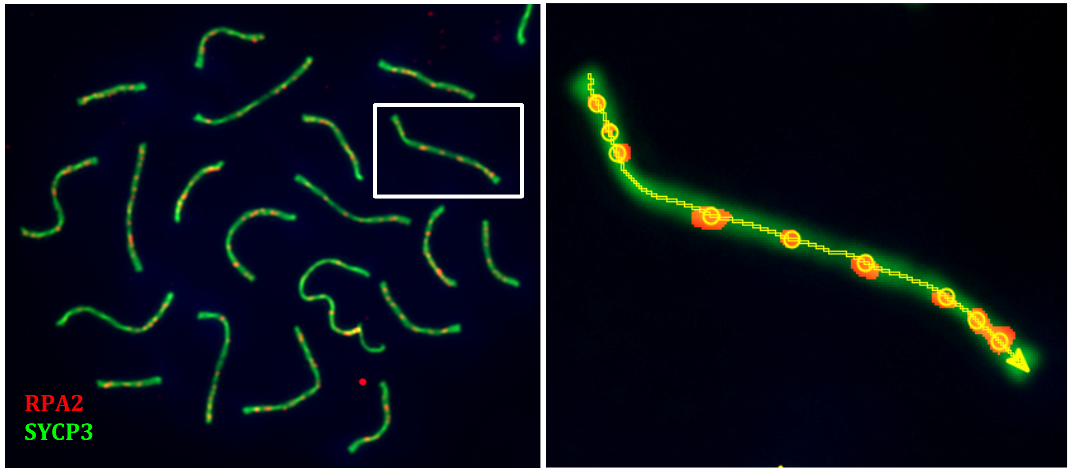

---SC---	Total length	COs number	Partial length-1	Partial length-2	Partial length-3	Partial length-4	Partial length-5	Partial length-6	Partial length-7	Partial length-8	Partial length-9	Partial length-10	Partial length-11	Partial length-12	Partial length-13	Partial length-14	Partial length-15	Partial length-16
SC-1	7.477	8	1.280	0.292	0.977	0.677	1.605	1.151	0.928	0.455	0.114	0.000	0.000	0	0	0	0	0
SC-2	11.043	8	1.136	2.582	1.090	2.463	0.825	1.036	1.203	0.503	0.206	0.000	0.000	0	0	0	0	0
SC-3	8.310	10	1.421	0.784	0.401	0.511	1.793	0.583	0.784	0.621	0.594	0.658	0.159	0	0	0	0	0
SC-4	5.508	8	1.144	0.682	0.776	0.511	0.575	0.492	0.905	0.174	0.250	0.000	0.000	0	0	0	0	0
SC-5	10.696	9	0.739	0.560	0.715	1.699	1.488	1.624	2.240	0.420	0.594	0.617	0.000	0	0	0	0	0
SC-6	7.348	11	0.096	1.332	0.530	0.284	0.651	1.574	0.605	0.348	0.575	0.720	0.428	0.205	0	0	0	0
SC-7	10.758	13	1.366	0.894	0.383	0.292	1.129	0.928	0.447	0.803	0.666	0.984	1.094	0.538	0.557	0.680	0	0
SC-8	10.992	12	0.744	0.484	0.594	0.401	0.110	0.329	1.290	0.810	3.112	1.616	0.455	0.647	0.399	0	0	0
SC-9	6.503	9	0.825	0.712	0.594	0.503	0.806	0.605	0.972	0.484	0.583	0.418	0.000	0	0	0	0	0
SC-10	10.373	8	1.120	1.880	2.054	1.325	0.701	0.883	1.102	0.647	0.662	0.000	0.000	0	0	0	0	0
SC-11	7.652	12	0.847	0.677	1.068	0.583	0.591	0.666	0.549	0.348	0.760	0.386	0.386	0.503	0.288	0	0	0
SC-12	10.326	11	1.382	0.447	0.773	0.519	0.401	0.787	1.494	1.276	1.582	1.074	0.431	0.161	0	0	0	0
SC-13	6.308	8	1.092	0.522	0.514	0.988	1.386	0.428	0.428	0.409	0.542	0.000	0.000	0	0	0	0	0
SC-14	8.542	8	1.318	1.147	1.075	0.310	1.347	1.192	0.696	0.814	0.644	0.000	0.000	0	0	0	0	0
SC-15	11.414	9	0.929	0.568	0.908	0.514	0.450	0.712	0.629	4.432	1.619	0.655	0.000	0	0	0	0	0
SC-16	10.714	8	1.938	1.814	2.801	1.249	0.447	0.511	0.674	0.920	0.360	0.000	0.000	0	0	0	0	0
SC-17	11.641	15	1.125	0.655	0.318	1.193	0.640	1.025	1.025	0.560	0.364	1.268	0.110	0.420	0.905	1.033	0.375	0.626
SC-18	4.991	4	1.655	1.019	1.220	0.409	0.689	0.000	0.000	0.000	0.000	0.000	0.000	0	0	0	0	0
SC-19	7.308	7	1.689	0.731	1.521	0.428	0.519	0.401	0.738	1.282	0.000	0.000	0.000	0	0	0	0	0
SUM	167.904	178																

H) House mouse (*Mus musculus*, courtesy of Parijat Chakraborty and Francesca Cole, The University of Texas MD Anderson Cancer Center, USA)

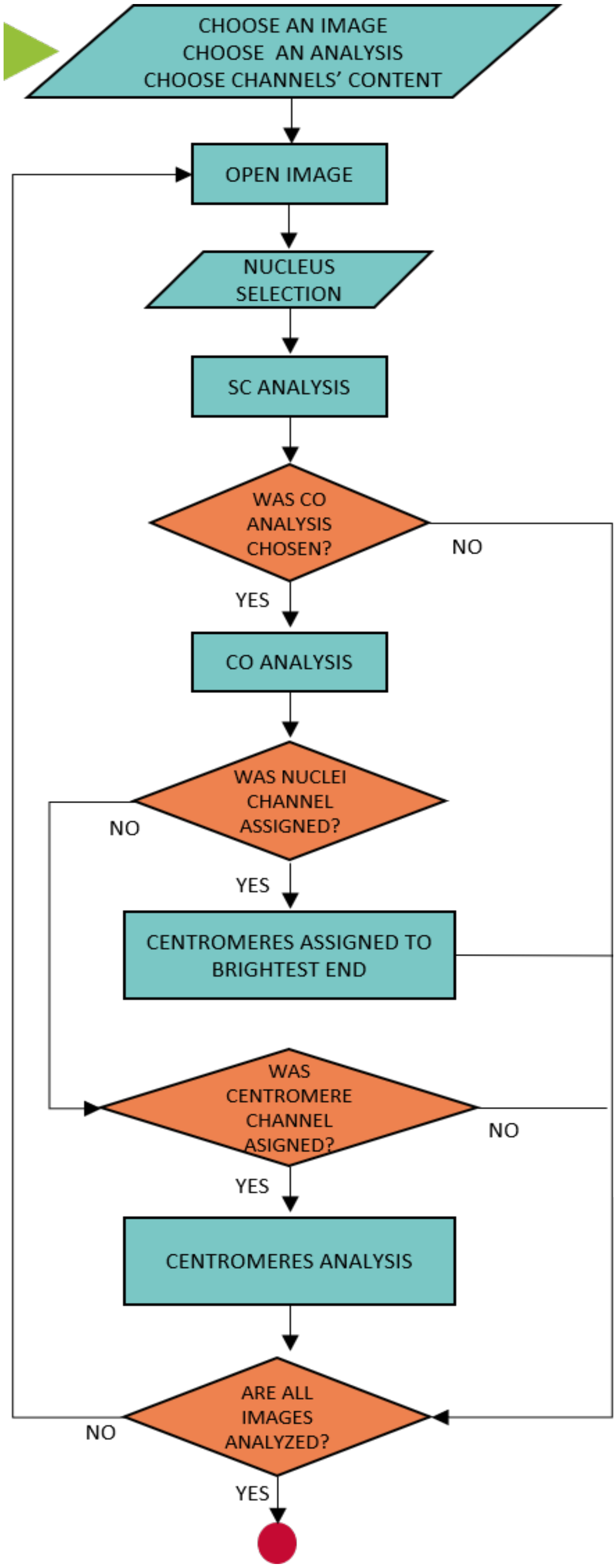

Supplementary Figure 2: Synaptonemal & CO Analyzer's process and validation results. Flow diagram of the general process.

The macro begins at the green triangle with a set of user graphic interfaces (GUI) that guide the user to select the first image for analysis, the analysis to perform (SCs only or SCs plus COs) and the content of each channel's image (SCs, nuclei staining, centromeres or COs). The main loop begins by selecting the nucleus to analyse in the opened image (at this point, sex chromosomes can be excluded from the selection if desired). SC analysis is always done, but CO analysis is performed only if selected. Centromeres' analysis is also optional: it can be performed based on DAPI signal intensity if a nuclei staining channel was introduced, or based on a specific signal if a centromere labelling (e.g., with CREST serum) channel was selected; when no centromere identification is performed, data will be analysed starting from the upper-left extreme of each SC. Once the analysis of an image is completed, the macro will automatically open the next one if more the one image is present in the same folder, until all are finished (red circle). Blue rhomboids represent GUI, blue rectangles depict actions and orange rhombus represent decision taking steps.

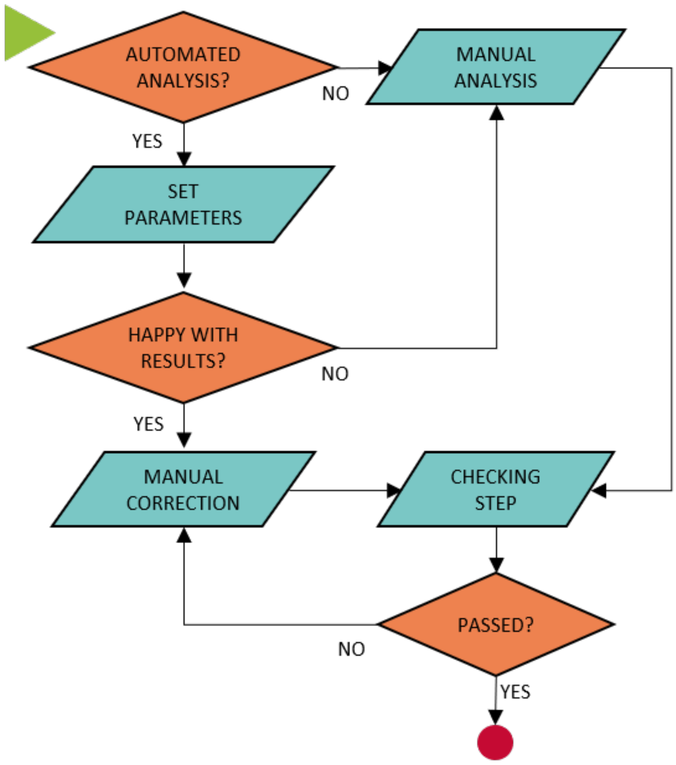

Supplementary Figure 3: Synaptonemal & CO Analyzer's process and validation results. Flow diagram of the semiautomatic process.

Although most of the analysis can be automatically performed, the user is given the choice for manual control at several steps. Analysis begins at the green triangle by asking the user whether to perform a manual or automated detection of SCs and centromeres. If automated analysis is chosen, the macro only requires from the user to set the intensity threshold for SC detection and to select background areas for COs (as shown in C) and centromeres identification (if applicable). With these parameters the macro automatically detects SCs and COs, but the user is given the opportunity of manually correcting minor mistakes -or opting for manual analysis. A final checking step verifies that SCs have been reduced to linear objects, COs and centromeres lay over SCs, each with no more than one centromere. If the verification fails, GUI guide the user to correct errors, while successfully verified images generate the final data (red circle).
