# Supplementary material for: Synaptonemal & CO analyzer: A tool for synaptonemal complex and crossover analysis in immunofluorescence images
Source: Front Cell Dev Biol. 2023 Jan 19;11:1005145. doi: 10.3389/fcell.2023.1005145 (PMC9894712; doi:10.3389/fcell.2023.1005145)
Supplement: Supplementary file 2 [file DataSheet1.PDF]

SYNAPTONEMAL & CO ANALYZER

ANALYZING SYNAPTONEMAL COMPLEXES (SCs) AND CROSSOVERS (COs)

Soriano et al, 2022

Index

1. GETTING STARTED: INSTALLING, UPDATING AND RUNNING THE MACRO.............................. 2
1.1 Installing ImageJ-Fiji 2
1. 2 Installing the Morphology Plugin Package 2
1. 3 Downloading the Synaptonemal & CO analyzer macro 2
1.4 Running the Synaptonemal & CO analyzer plugin 2
2. GENERAL CONSIDERATIONS ON THE OPERATION OF THE MACRO 3
3. WORKING WITH SYNAPTONEMAL & CO ANALYZER: ANALYSIS OF SCs and COs. 7
3. 1. Run the macro. 7
3.2. Select the images' folder. 7
3.3. Select an analysis... 7
3.4. Enter the content of each channel. 8
3.4. Nucleus selection. 9
3.5. Intensity-based SCs selection. 10
3.6. Smooth manual SCs selection. 11
3.7. Automatic SCs detection. 16
3.8. Automatic COs detection. 17
3.9. Manual COs selection correction. 18
3.10. Automatic COs examination... 20
3.11. Automated analysis. 20
4. SYNAPTONEMAL & CO ANALYZER: RESULTS. 20
4.1. Importing results to Excel.. 22
5. WHATS NEW ON SYNAPTONEMAL & CO ANALYZER 2.0? 24
5.1 SYNAPTONEMAL & CO ANALYZER 2.0 opens a wider variety of image formats 24
5.2 SYNAPTONEMAL & CO ANALYZER 2.0 eases adapting the macro to your own images ... 24
5.2.1 Users with neither imageJ programming nor image analysis skills........................... 25
5.2.2 Users with image analysis experience.. 26
5.2.3 Users with imageJ macro language programming skills. 27
5.3 SYNAPTONEMAL & CO ANALYZER 2.0 is able to analyze centromeres' labeled images .. 27
5.4 SYNAPTONEMAL & CO ANALYZER 2.0 eases manual COs and centromeres detection and checking automatically detected results 30

1. GETTING STARTED: INSTALLING, UPDATING AND RUNNING THE MACRO

1.1 Installing ImageJ-Fiji

Go to https://imagej.net/Fiji

Choose your operative system, download FIJI and extract the obtained compressed folder. Double click on ImageJ-win64.exe to start FIJI. Take note of the location of FIJI's folder.

iOS users: installation permission should be granted in Systems Preferences> Security & Privacy> General> Open anyway> Open. FIJI must be located in the Applications folder in order to be able to install updates and plugins.

1. 2 Installing the Morphology Plugin Package

Open Fiji and select on the menu bar: Help> Update …> Manage update site. In the pop-up menu select the checkbox "Morphology", then click on the buttons "close" and "Apply changes".

Go to https://imagej.net/ImageJ_Updater for further documentation.

1. 3 Downloading the Synaptonemal & CO analyzer macro

Go to https://github.com/joaquim-soriano/Synaptonemal-and-CO-analyzer and click on Code>Download ZIP. This will produce a .zip folder containing a read me and a license file, test images, a user's manual, a video tutorial and the macro (Synaptonemal_&-CO_analyzer.ijm).

Windows users: make sure FIJI is not running (close the program if necessary) and copy and paste the macro into FIJI's plugins folder (Fig.1, top). iOS systems users: copy and paste the macro to FIJI's plugins folder (Fig.1, top), open Fiji, select on the menu bar: Plugins>Install…, select the previously downloaded file (Fig.1, middle) and click Save. Then restart FIJI.

1.4 Running the Synaptonemal & CO analyzer plugin

Start FIJI, click on the Plugins menu on the menu bar, then click on Synaptonemal & CO analyzer to run the application (Fig.1, bottom).

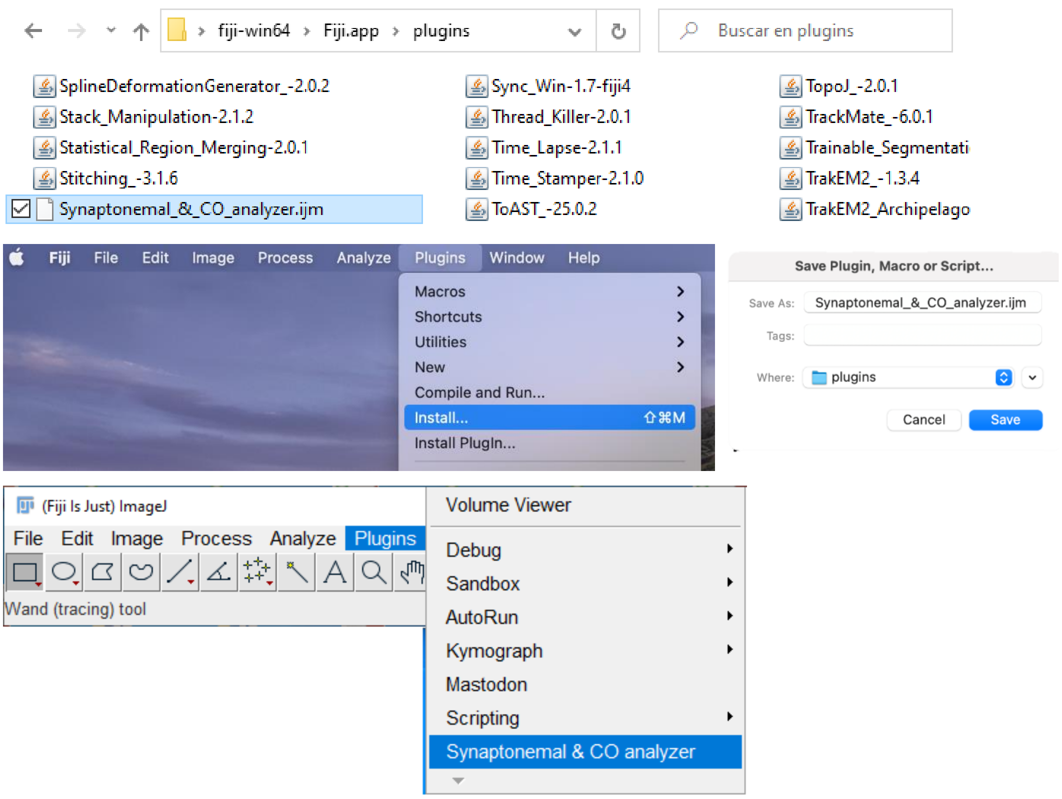

Figure 1. Top: plugins' folder. Downloading the *Synaptonemal_&_CO analyzer.ijm* file to this folder is the easiest way for windows users to install the macro. Middle: iOS users need to install the macro via FIJI's Plugins Menu. Bottom: once installed, the macro can be executed from the Plugins Menu.

To update the plugin, repeat the steps above replacing the oldest version by the new one.

2. GENERAL CONSIDERATIONS ON THE OPERATION OF THE MACRO

• The macro is intuitive and easy to use. FIJI's regular users may make it work without any further reading. We strongly recommend itchy users to try the video tutorial in: https://github.com/joaquim-soriano/Synaptonemal-and-CO-analyzer

• Once executed, the macro will guide the user through consecutive steps via popup windows filled with text instructions and action boutons. Occasionally, the user will be asked to interact with images to perform a task (checking automatically detected crossovers, for example). Most FIJI's commands can be accessed meanwhile, bear in mind that inappropriate use of this feature can cause undesired results (e.g., closing the image under analysis will cause the macro to fail in a following step).

• The macro will consecutively process all images in a folder. Images to analyze must be of the same kind (*i.e.*, have the same file extension). They should also have the same channels' number and distribution, otherwise the macro could deliver unexpected results. Non-image files, subfolders and images with different extensions on the folder under analysis will not have any effect.

• The macro will work on TIFF, GIF, JPEG, BMP, PNG, PGM, FITS and ASCII images.
• The macro will not work on ·3D images. Users willing to analyze images with different planes need to collapse them on a single one. This might cause COs to overlap, it might also introduce changes on SCs' length and shape. The user should inspect the images to tell whether these changes occur or are irrelevant. Use the ZProject tool on the image menu (https://imagej.nih.gov/ij/docs/menus/image.html#stacks) to collapse z planes using imageJ/FIJI.

• The macro works on multichannel images. Channels' content determines the analysis that can be performed, as shown in table 1.

Minimum number of channels	Channel's content	Analysis
1	SCs	SCs length
2	SCs, DAPI	SCs length
2	SCs, COs	SCs length, COs positions relative to a random SC end
3	SCs, COs, DAPI	SCs length, COs positions relative to the more DAPI intense SC end

Table 1: minimum number of channels per image to perform each meiotic recombination analysis. Extra channels need not to be used, i.e., SCs length only analysis can be performed on three channel images.

Three channel images allow for conducting all meiotic recombination analysis, two channel images allow for SCs and COs analysis, at least one channel is needed for the macro to work. A DAPI channel is used for aiding the user to locate nuclei and telomeric centromeres (in some species, i.e., mouse, the centromere is always at one chromosome end and is more intense for DAPI staining).

• Results will be automatically saved in a "Results" folder in the source images folder. This folder will be overwritten if Synaptonemal & CO analyzer is run twice on the same images folder, should this be the case, the macro would create a warning and give the user the chance to rename or relocate the "Results" folder prior to overwriting it.

• The macro can be interrupted anytime by selecting a window and pressing the "i" key on the keyboard.

• FIJI's magnifying glass and scrolling tool can be used to better edit and analyze images. Use the magnifying glass to zoom in (left click) and zoom out (right click) the image. Use the scrolling tool to move through an amplified image (the scrolling tool can be activated anytime by pressing the space bar on the keyboard). Follow this link for more info on FIJI's tool bar: https://imagej.nih.gov/ij/docs/tools.html.

• It is not possible to revisit all previously executed steps. However, checkpoints are established on critical decisions and the opportunity is given to correct mistakes and modify automatic detection results (e.g., automatically detected SCs can be manually shortened, elongated or trimmed afterwards).

• The "Results" folder contains a subfolder per each analyzed image named after it. The following files are created (Fig.2):

  - A .tif multichannel image of the analyzed nucleus (Fig.2, top left).

- A text file containing a table with all measured distances on each SCs. This table contains SCs total length and partial centromere and COs defined distances (in the event that COs have been analyzed Fig.2, bottom).This file can be imported into Excel or any other spreadsheet for analysis.
- Compressed .zip folders containing coordinates for the detected nucleus, COs, SCs and centromeres positions (or SCs starting points, if no DAPI channel has been selected). These folders allow for visualizing detected structures over the analyzed image. If willing to check results, simply open the analyzed image and drag and drop any of these files to FIJI's main window to overlay the desired structures (Fig.2, top left). This visualizing tool depends on a FIJI's function named ROI Manager. To highlight a specific structure, select it on the ROI manager panel (Fig.2, top right), click this link for further reading on how to use this tool: https://imagej.nih.gov/ij/docs/menus/analyze.html#manager.

Those files allow for checking results any time after the analysis is done.

• The XY chromosome is excluded from most meiotic recombination studies. Should this be the case, either exclude its SC from the nucleus selection at step 3.4 (Fig.7) or erase it at step 3.6 (Fig.10).

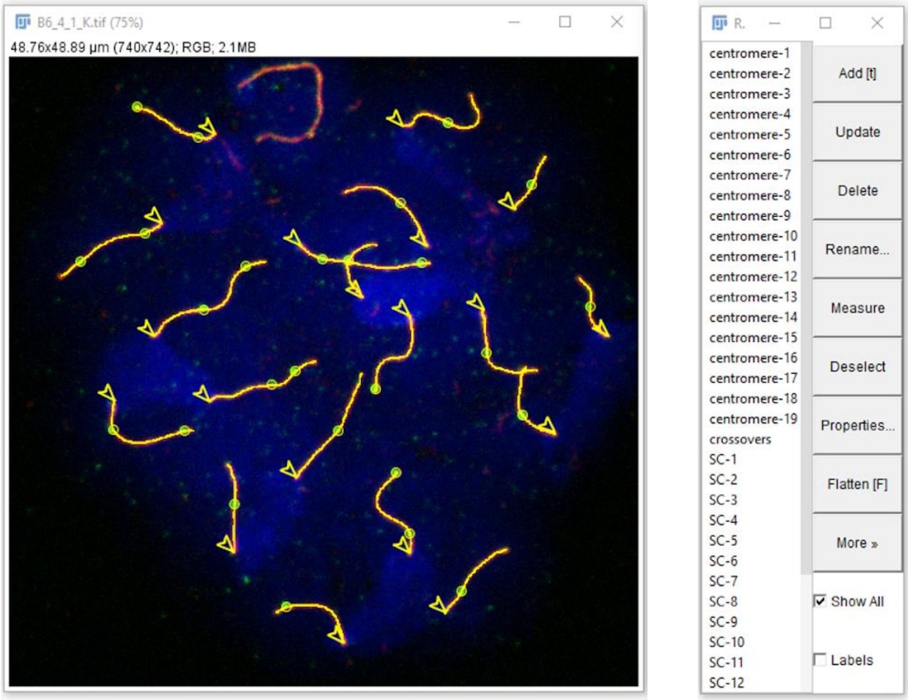

SC's length_B6_4_1_K.lsm.txt

File Edit Font

	---SC---	Total length	COs number	Partial length-1	Partial length-2	Partial length-3
1	SC-1	9.358	1	4.296	5.062	0.000
2	SC-2	7.409	2	1.503	5.766	0.140
3	SC-3	5.186	1	2.462	2.724	0.000
4	SC-4	9.186	1	4.187	4.998	0.000
5	SC-5	9.561	2	1.626	5.909	2.026
6	SC-6	12.067	2	4.935	5.420	1.712
7	SC-7	5.713	1	3.017	2.696	0.000
8	SC-8	8.095	1	7.889	0.206	0.000
9	SC-9	9.635	2	5.400	2.359	1.876
10	SC-10	10.273	1	5.074	5.198	0.000
11	SC-11	9.628	2	2.517	6.431	0.681
12	SC-12	7.237	1	3.860	3.378	0.000
13	SC-13	8.538	2	1.658	6.834	0.047
14	SC-14	7.475	1	2.199	5.276	0.000
15	SC-15	7.540	1	6.553	0.987	0.000
16	SC-16	6.295	1	3.426	2.869	0.000
17	SC-17	10.996	2	2.196	8.174	0.626
18	SC-18	7.333	1	3.149	4.185	0.000
19	SC-19	8.537	1	3.571	4.966	0.000
20	SUM	160.064	26	----	----	----

Figure 2. Top left: .tif results image. Nucleus, COs, SCs and centromeres are overlayed. Top right: ROI manager allows to select each centromere, SC, nucleus and COs. Bottom: Results table showing the number of COs per SC, SC's total length, distance between the centromere and the first CO (Patial length-1), distance between the first and second COs (Partial length-2), etc. Partial lengths are only calculated if the user performs a CO analysis, otherwise only SCs length are computed.

3. WORKING WITH SYNAPTONEMAL & CO ANALYZER: ANALYSIS OF SCs and COs.

3. 1. Run the macro.

Click on Synaptonemal & CO analyzer on the Plugins menu to execute the macro (Fig.1 bottom) then follow the instructions on the following windows. Upon starting, the macro will automatically close all FIJI open windows (Fig.3) nonetheless, the user has the chance to save any previous unsaved data.

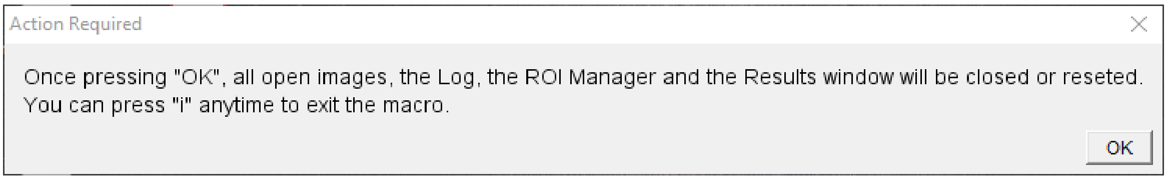

Figure 3: executing Synaptonemal & CO analyzer re-initializes FIJI, but the user is warned to save valuable data before.

3.2. Select the images' folder.

Choose an image in the folder containing the images to be analyzed (Fig.4). Make sure that this folder meets the requirements described on the "General Considerations" section.

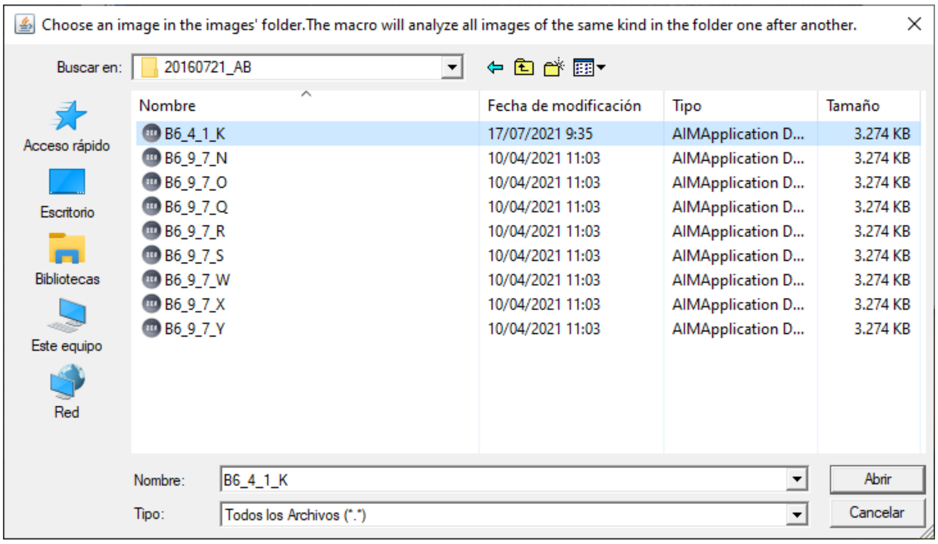

Figure4: If choosing the selected image, all images in the folder with the same file extension will be consecutively processed.

3.3. Select an analysis.

Choose whether to compute SCs' length and inter COs and centromere distances or SCs' lengths only (Fig.5). If the first option is chosen, the macro will continue on step 3.4, otherwise the macro will skip steps 3.4 to 3.7 and directly execute step 3.8.

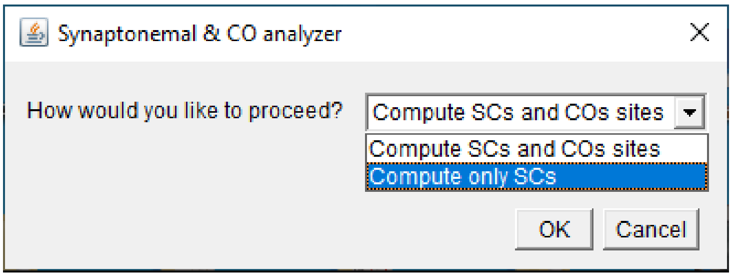

Figure 5: the user can choose whether to compute SCs' lengths only or SCs' length and COs distances. If a DAPI image is chosen later on, COs distances will be given relative to the most intense DAPI end (i.e., the centromere in mice), otherwise, COs distances will be computed from a random SC end.

3.4. Enter the content of each channel.

Enter the contents of each channel. For instance, enter 2 on the field "SCs are in image:" if the second channel of a multichannel image is containing a SCs staining (Fig.6). Channels contents are shown in a separate image (Fig.6 bottom). This menu is versatile, it won't ask for COs if "Compute only SCs" option is chosen in the previous menu (Fig.5) and it won't pop up if a single channel image is being analyzed (under this situation, the only possible analysis is a SC length analysis, consequently the macro will assume that the single channel image is containing SCs).

If the field "Nuclei are on image: "is set to 0, COs positions will be given relative to one SC end at random. Otherwise, the selected channel will be used to compute the SC end with the most intense DAPI staining (*i.e.*, centromeres in mice).

Meaningless options will deliver an explanatory error and the menu will pop up again so the user can introduce correct values.

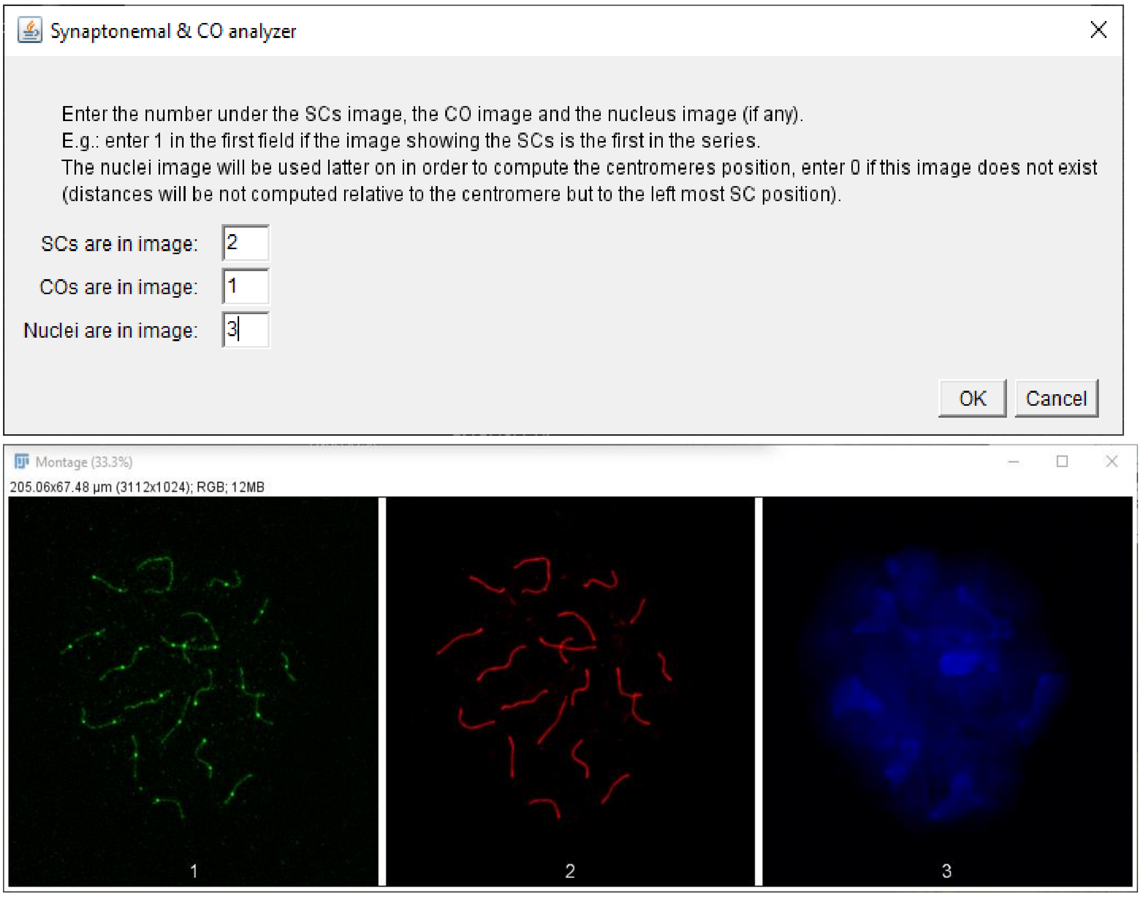

Figure 6: Selecting the SCs, COs and nuclei channel (if any). Top: when analyzing SCs and COs, the number under the image containing SCs, COs and nuclei is asked to be introduced. Bottom: A preview of the different channels of the image to be analyzed is shown to facilitate the operator's task.

3.4. Nucleus selection.

The macro automatically opens the first image on the images folder. Create a selection surrounding the area containing the SCs' to analyze using the more suitable channel (Fig.7). Click the "Add (t)" button on the ROI Manager and click "OK".

By default, the polygon selection tool is selected, however, the user can use any selection tool on FIJI. If no closed selection is done a following menu will offer the opportunity to either create a new one or analyzing all SCs in the image.

Follow this link for more info on how to use FIJI's selection tool: https://imagej.nih.gov/ij/docs/tools.htm

Tip, leave the XY chromosome SC out of the selection if possible, so you don't have to erase it afterwards.

An image containing different nucleus or groups of chromosomes needs to be analyzed twice, selecting each different group on subsequent analysis.

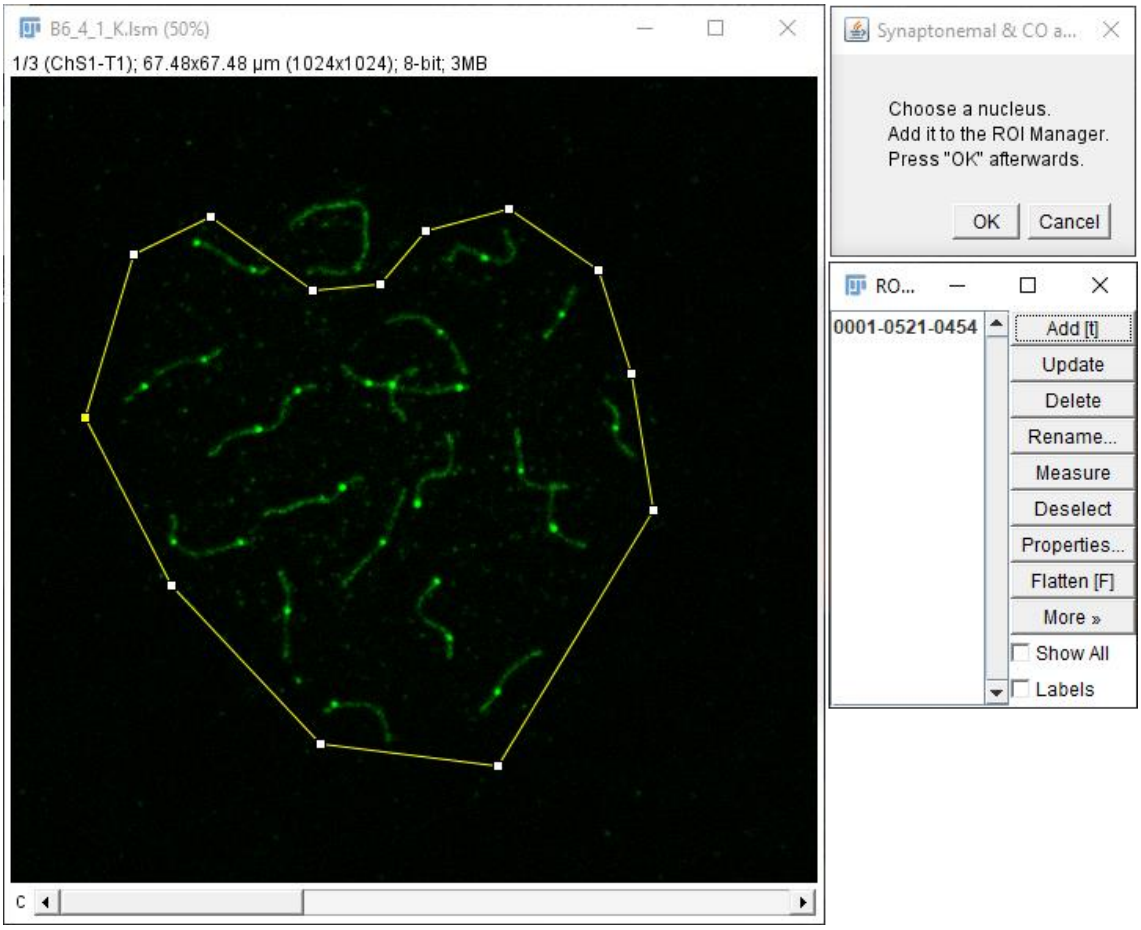

Figure 7: only SCs inside the selection will be analyzed. Note that the sex chromosome is out of the selection to avoid deleting it afterwards. Use the bar in the lower part of the image to visualize the channel best suited for this task. The selected area has already been added to the ROI Manager (right bottom).

3.5. Intensity-based SCs selection.

A black and white image containing the SCs automatically opens together with the Threshold tool as shown in *Fig. 8, Top*. Use the scroll bar to highlight the SCs trying to get rid of as much background as possible while keeping SCs's signal (*Fig. 8, Bottom*). Follow this link for more info regarding the Threshold tool: https://imagej.nih.gov/ij/docs/menus/image.html.

Follow instructions and select OK, do not click on "Apply".

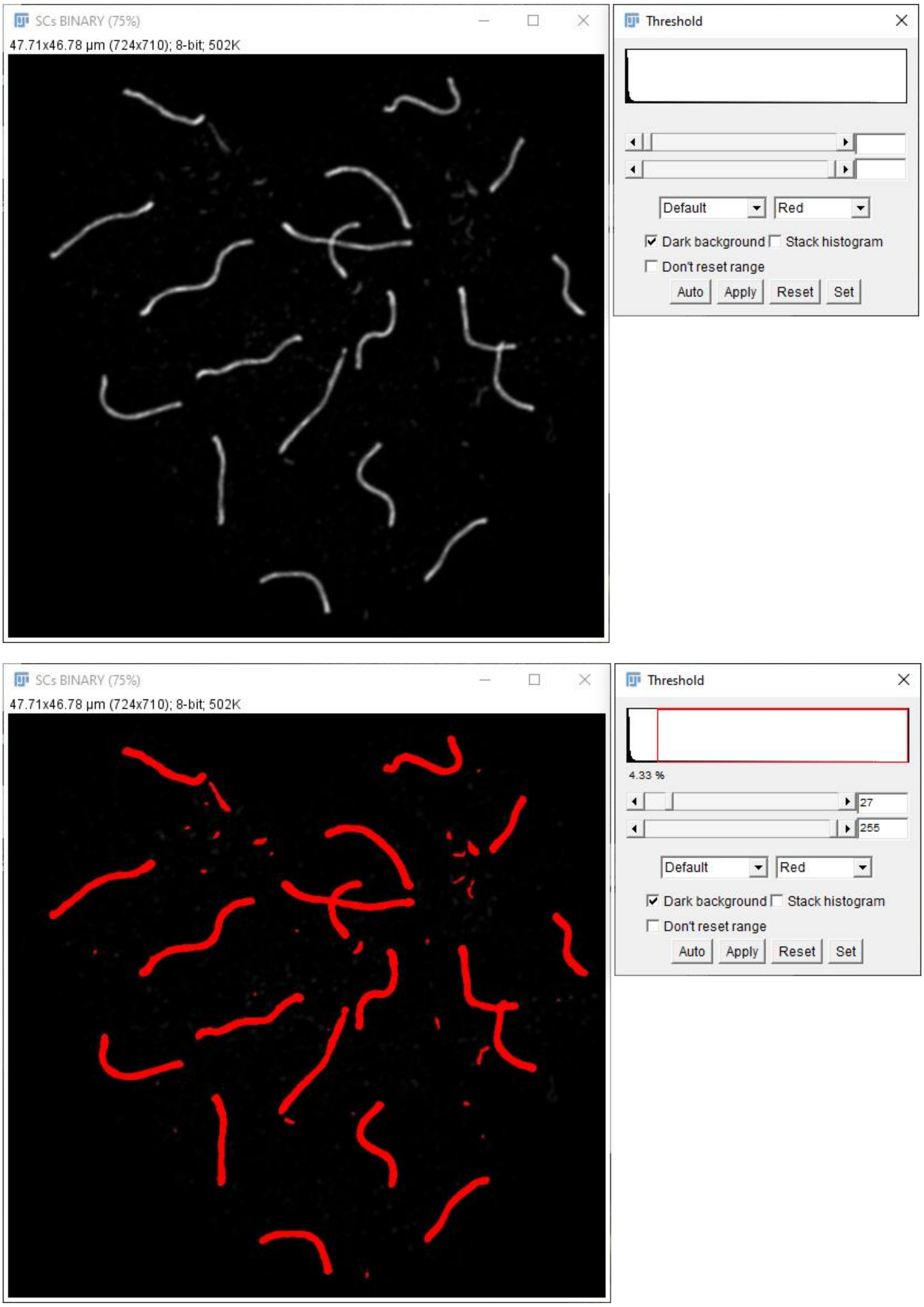

Figure 8: SCs are selected by means of its intensity using the Threshold tool. Top: Image prior to the threshold adjustment. Bottom: Image after the threshold adjustment using the tool showed on the right side.

3.6. Smooth manual SCs selection.

Once pressing OK, two images containing SCs pop up. One of them (“SCs BINARY”) shows a single pixel width representation of the selected SCs in black and white. The other image (“SCs 8-BIT”) shows the original image for comparison purposes (Fig. 9, bottom and Figs 10 to 12).

Press on "Synchronize All" and process the SCs on the black and white image until it represents a pixel width line of the SCs on the color image (Fig.9, top left and Figs 10 to 12).

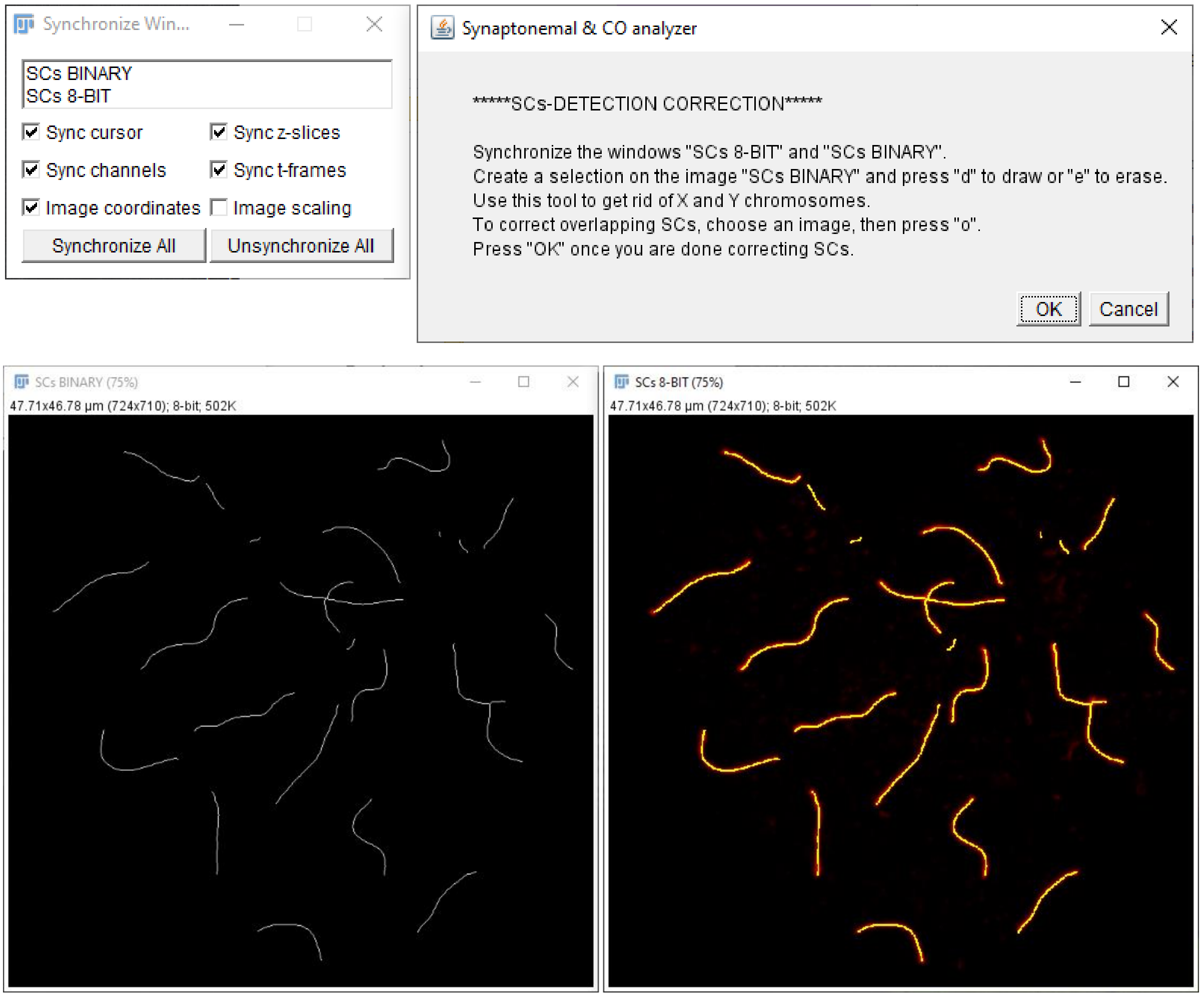

Figure 9: intensity-selected SCs can be corrected before analysis by using a set of tools. The bottom left image shows a single pixel representation of selected SCs in white, the bottom right image shows selected SCs in yellow over the original image. Both images must be synchronized (top left) so zoom and selections drawn in any of them are shown in both images at the same time.

Editing instruction are shown, briefly:

- Erasing: choose a selection tool on FIJI, create a selection over the region to erase then press "e" on the keyboard (Fig.10). Use this tool to erase the XY chromosome SC if necessary. The "Rectangle" selection tool is recommended for fine SCs pruning while the "Polygon" selection tool works better for entire SCs deletion.

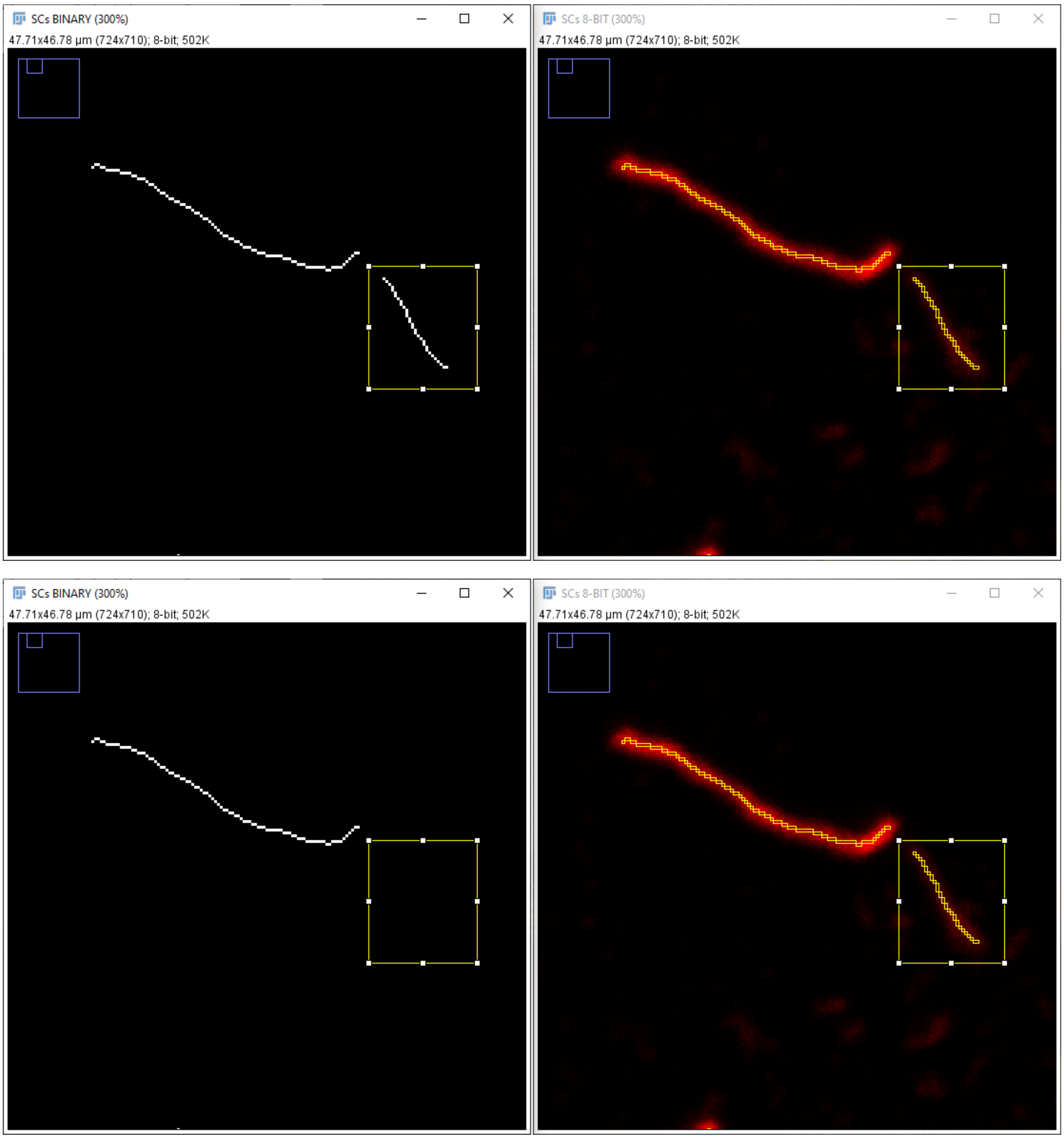

Figure 10: Manual removal of a signal erroneously detected by the macro using the "rectangle" tool. Top: erroneously detected SCs, notice rectangle tool selection prior to erasing. Bottom: corrected SCs.

- Drawing: choose a selection tool on FIJI, create a selection over the region to draw then press "d" on the keyboard (Fig.11). The "Freehand", "Straight"- and "Segmented-line" tools are recommended (follow this link for more info on how to use them: https://imagej.nih.gov/ij/docs/tools.html

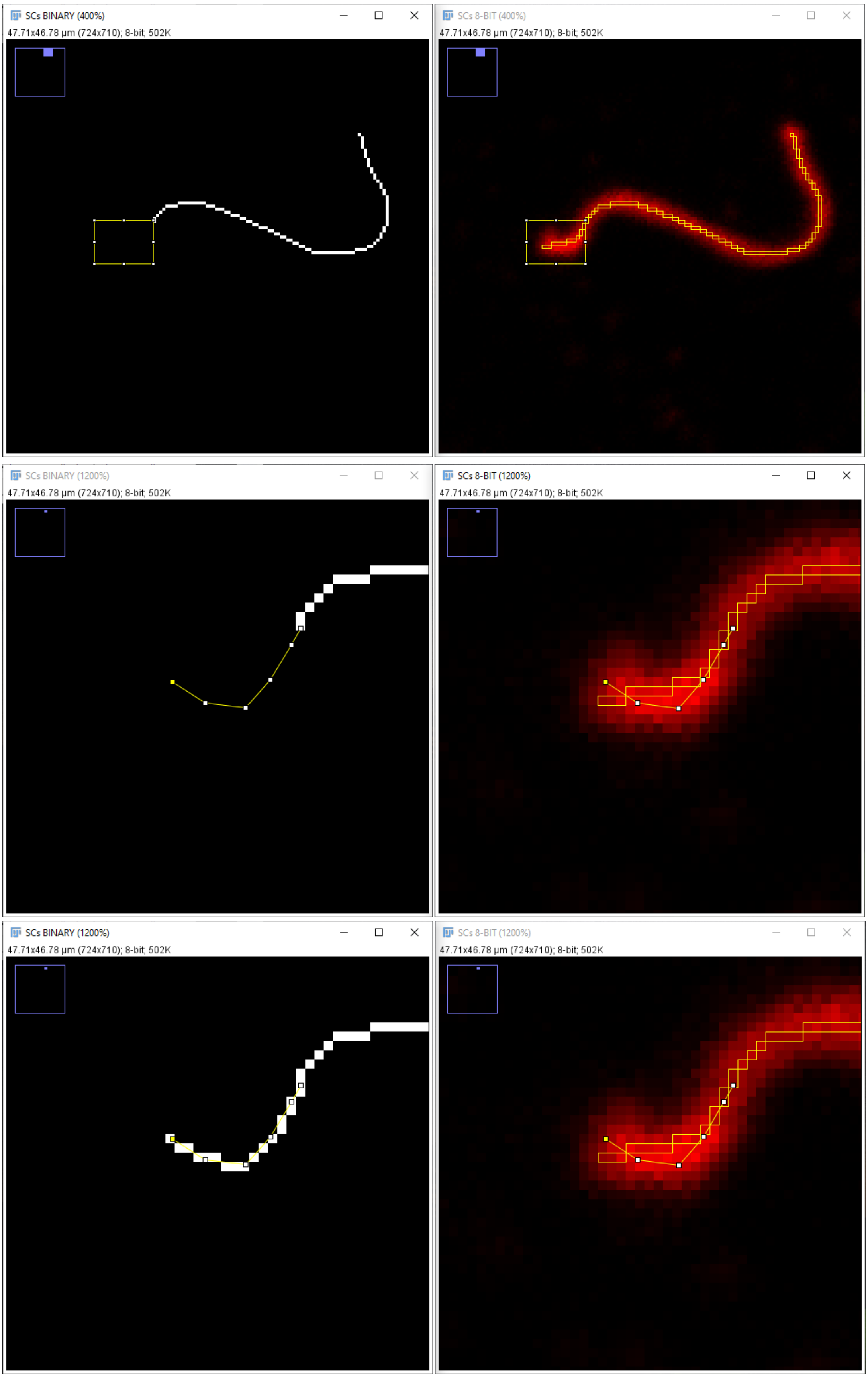

Figure 11: Manual drawing of a signal badly detected by the macro using the " Segmented-line " tool. Top: a part of the SC that is seen on the right image is missing on the left image. Middle: segmented-line selection prior to drawing. Bottom: newly drawn, corrected signal.

- Correcting overlapping SCs: choose an image, press “o” on the keyboard and follow instructions. 1- Enter the number of overlapping SCs and create a selection containing the overlapping chromosomes (Rectangle or Polygon selection tools recommended) (Fig. 12, top). 2- A pop-up window will instruct the user to draw one of the overlapping SCs, do it by using the erasing and drawing tools above. Press “OK”, then repeat the same procedure for the remaining overlapping SCs (Fig. 12, bottom).

Please notice that two windows with instructions and an “OK” button remain open in this step, make sure to press the “OK” button in the last appearing window immediately after following the directed instruction, the macro will end up and deliver an error message otherwise.

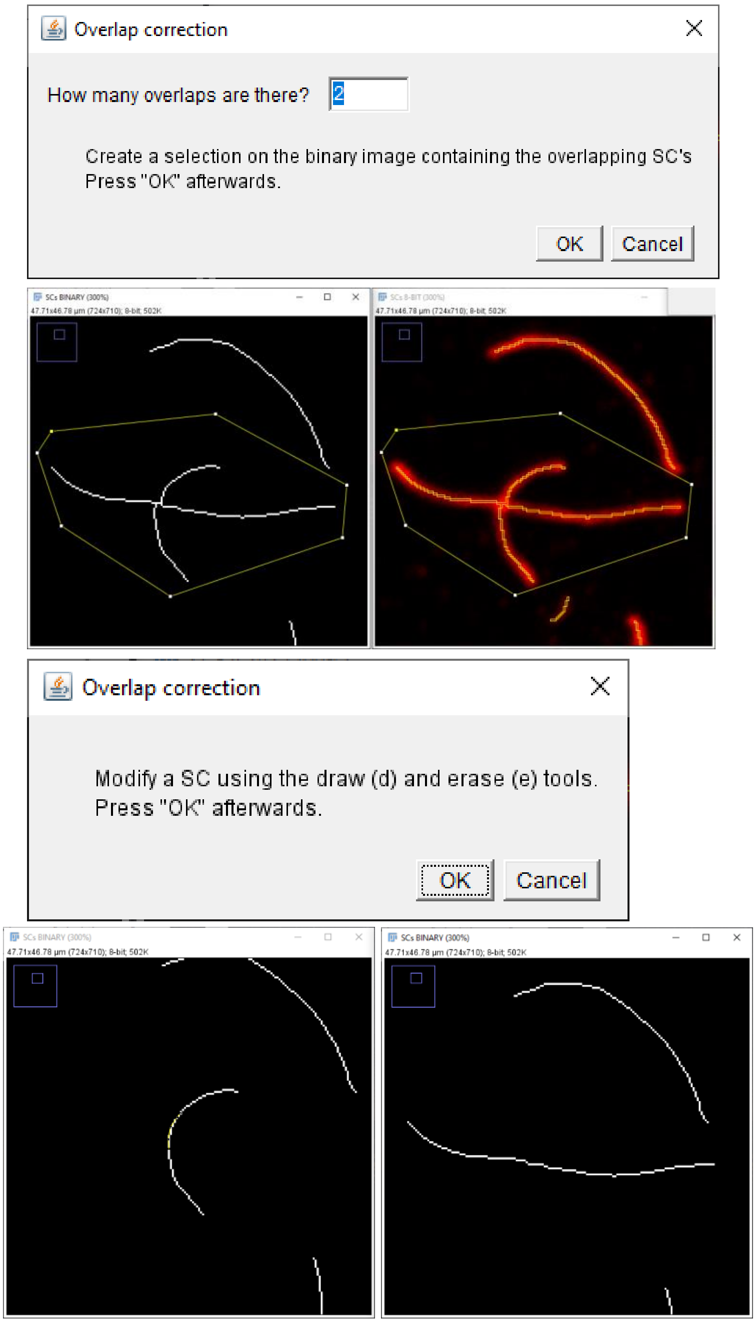

Figure 12: Correcting overlapping SCs. Press the "o" key and do as instructed. Once the number of overlapping SCs are introduced and a selection surrounding them done (upper images and menu)), the lower menu pops up as many times as needed to allow for drawing individual SCs (bottom).

Once all needed modifications have been made, press the "Ok" button shown in Fig.9 top-right.

3.7. Automatic SCs detection.

The macro automatically checks the SCs for branching points. If any of them were detected the user is redirected to step 6 to get rid of them by using the aforementioned editing tools. SCs are numbered to facilitate its detection (Fig, 13).

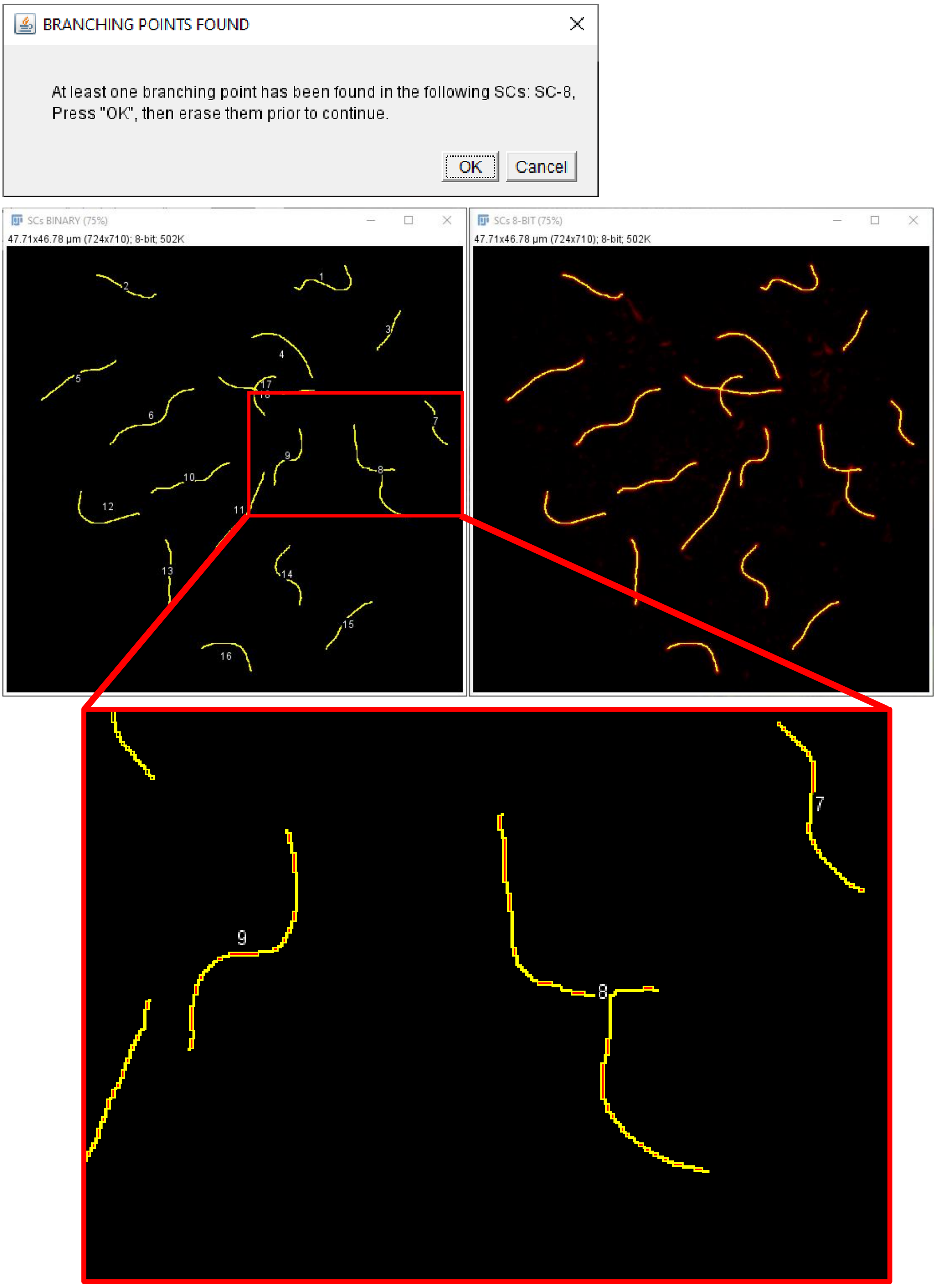

Figure 13: Branching points are automatically detected. The user is directed to them for editing.

The macro will finish here by automatically analyzing selected SCs in case "Compute only SCs" was selected on step 3.3, otherwise step 3.8 will be executed.

3.8. Automatic COs detection.

Choose whether trying to locate COs automatically or locate them manually (*Fig. 14)*

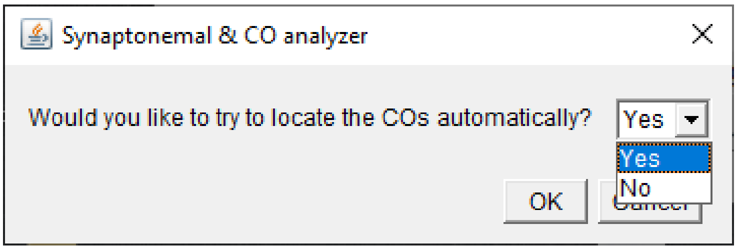

Figure 14: the user can choose whether to locate COs automatically or manually.

To locate COs automatically, create some selections over the SCs background, it should contain fragments of SCs that lack COs. Press the shift key on the keyboard to do different selections (*Fig.15*). The Polygon selection tool is recommended.

Tip: the automatic detection routine works better if SCs' fragments with a fairly bright background are selected.

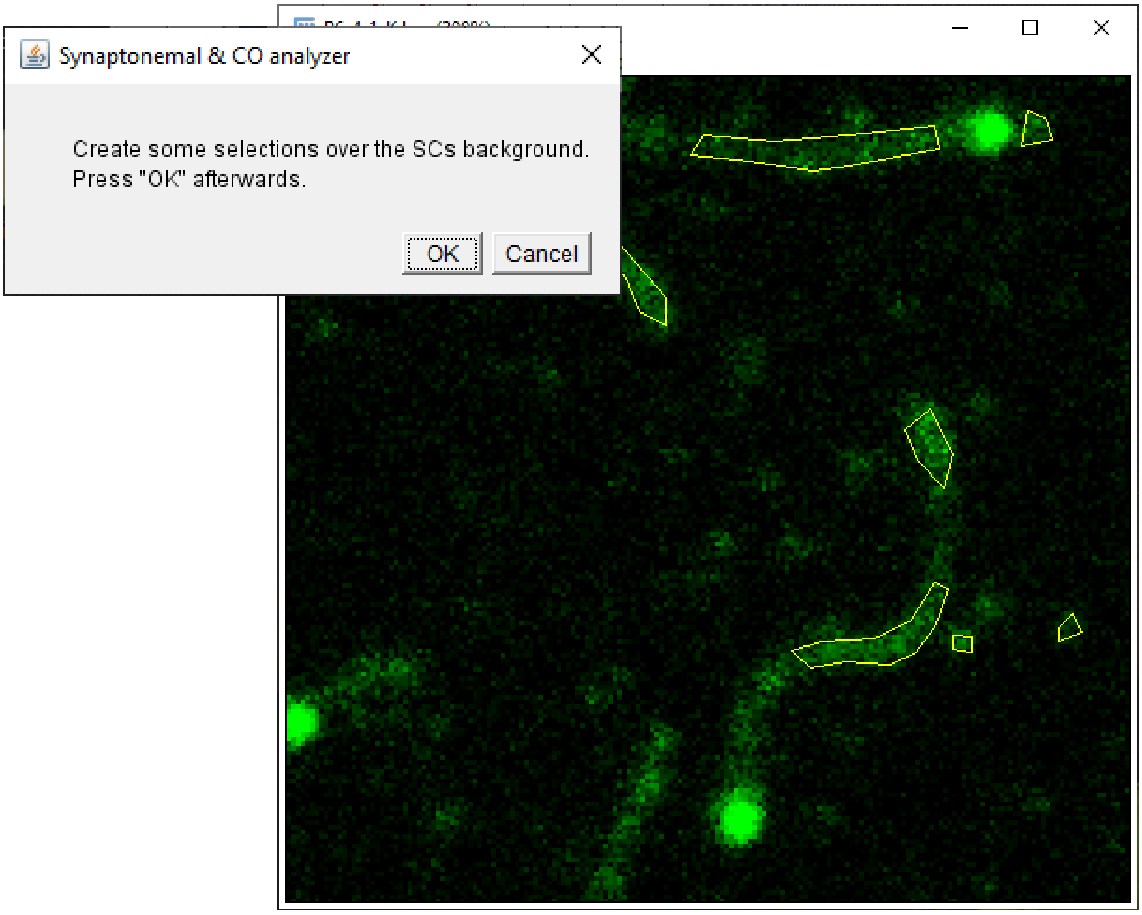

Figure 15: Automatic COs selection. Choose background signal by using the Polygon selection tool. Do not select COs nor parts of the image without any background at all.

Press OK, then check the automatically selected COs (Fig.16, top) and choose whether to continue correcting minor errors manually or locating COs manually (Fig.16, bottom).

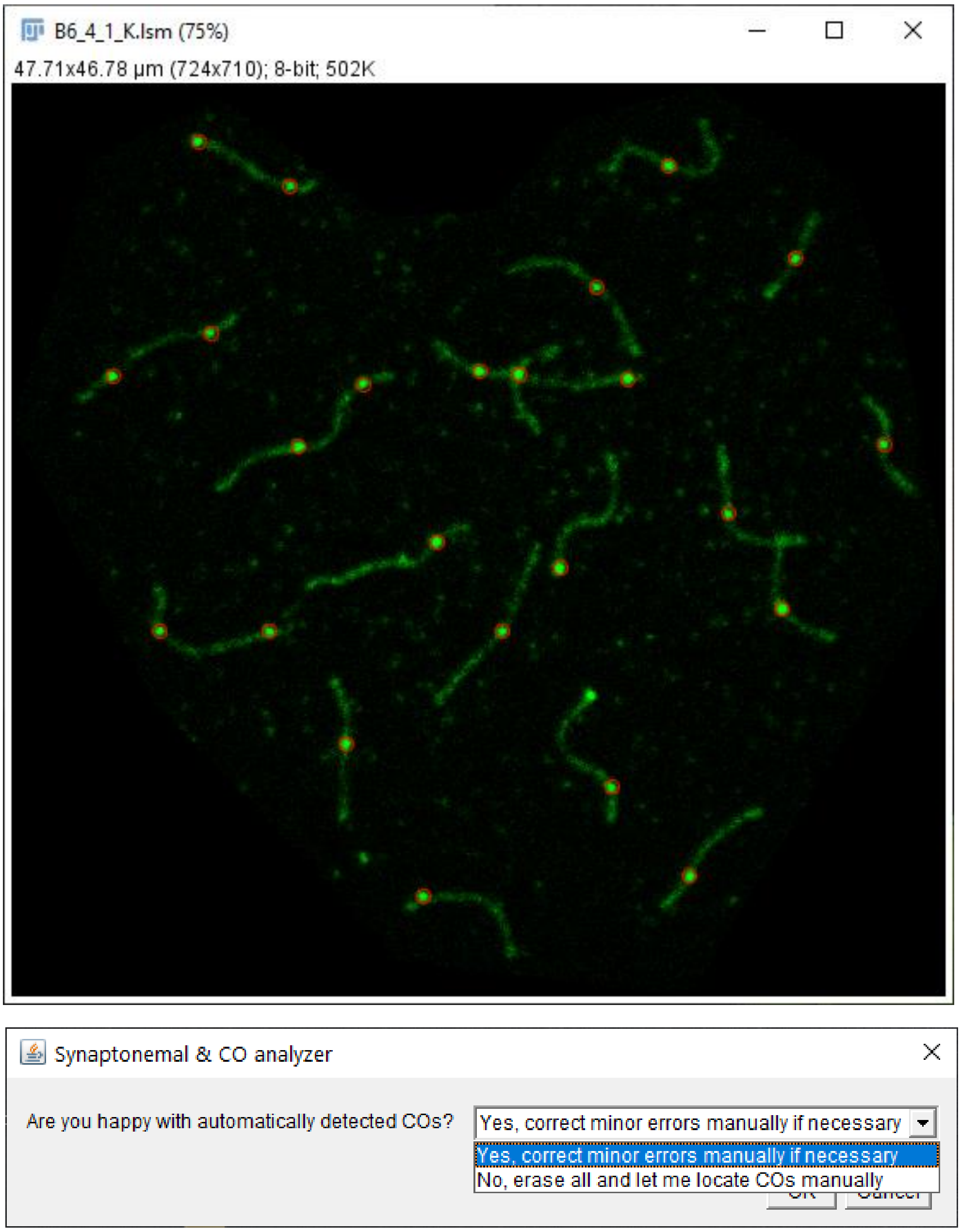

Figure 16: automatically detected COs are shown as red circles (top). If happy with results, minor errors can be corrected manually, otherwise COs can be selected manually (bottom).

3.9. Manual COs selection correction.

Once clicking OK, the macro carries out an automatic COs foci detection and the results are shown over the original COs image (Fig.17, bottom). Correct any mistakes as instructed (Fig.17, top):

a. Left click on any location to add a CO.
b. Hold down the "Alt key" and left click on a CO to delete it.
c. Right click over a CO and drag and drop to change its location.

Make sure that the Multipoint selection tool is selected during this process (although automatically selected, it is sometimes left unselected after zooming in/out the image, which we strongly recommend).

The same instructions above should be followed in case a manual COs detection is done instead of an automated one.

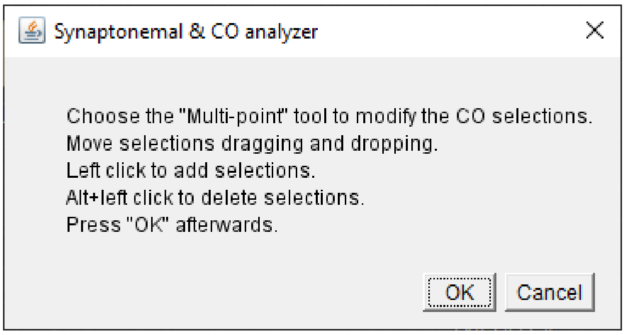

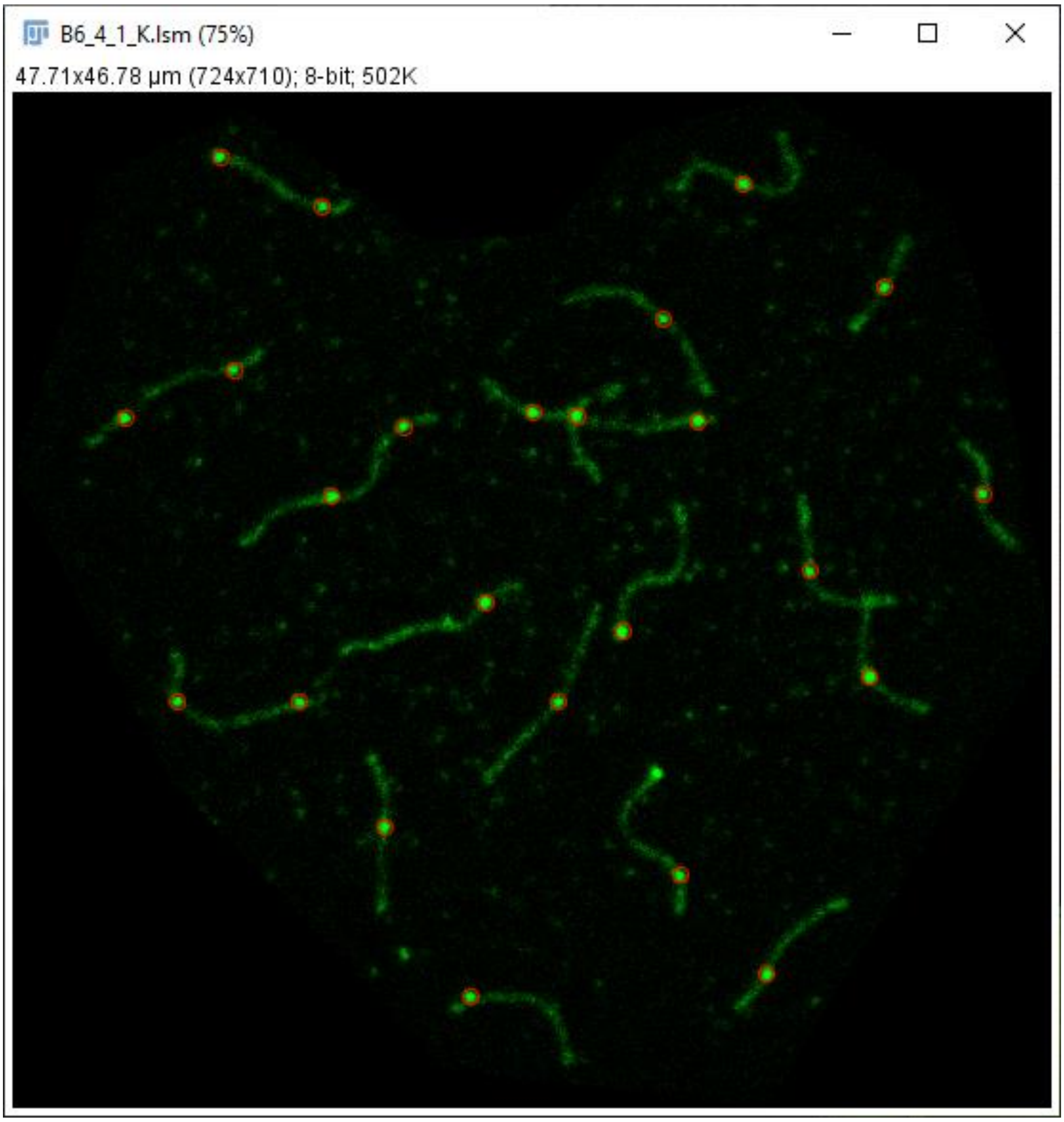

Figure 17: Automatically detected COs (red circles on the original COs image) can be added or erased manually according the given instructions (Top). If no automatic analysis is done, all SCs must be added manually.

3.10. Automatic COs examination.

The macro automatically assigns each CO to the closest SC, if failing to do so, the troublesome CO is indicated and the user is instructed to move it to the closest SC (Figure 18).

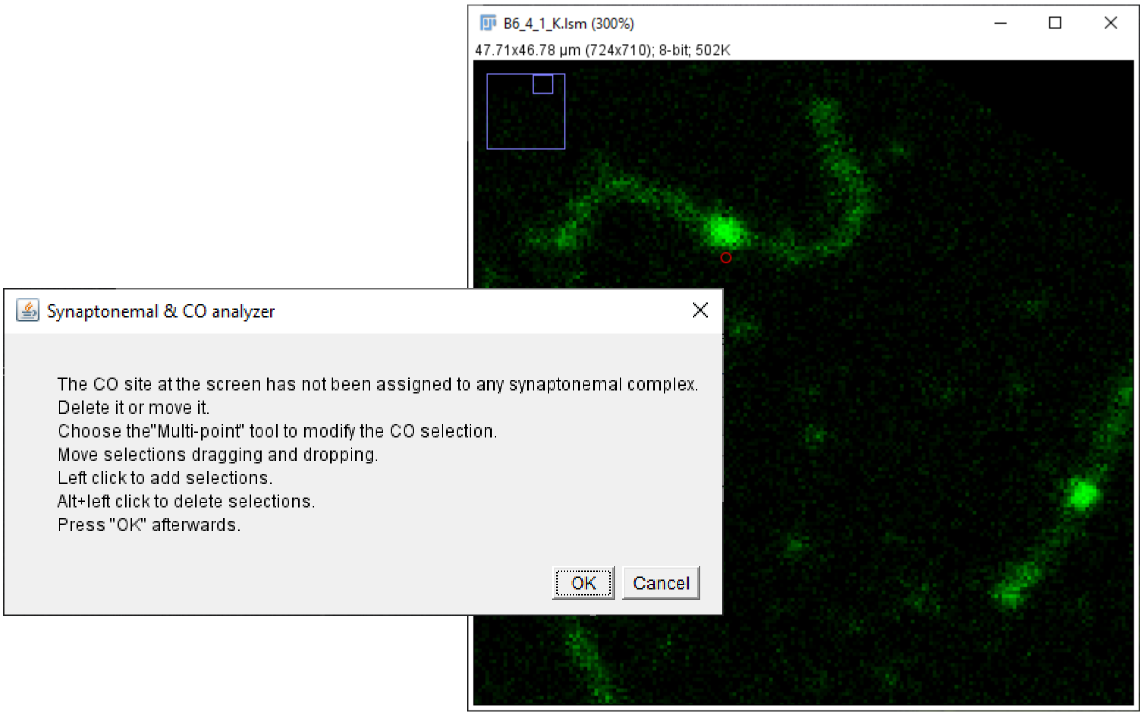

Figure 18: If any automatically detected CO cannot be assigned to a SC (right), the macro will indicate the user to move it to the closest SC following the given instructions (left)

3.11. Automated analysis.

The macro automatically detects the centromere on every SCs and analyzes the length of every inter CO distance starting from this point. Results are automatically saved as told in the General Consideration section.

Steps 3.4 to 3.11 are repeated for every image in the images folder. Once all the images in the folder have been analyzed, the macro will display a completion message.

4. SYNAPTONEMAL & CO ANALYZER: RESULTS.

Once the analysis is done, a subfolder named “Results” is created inside the folder that contained the original images. Inside the results folder, a subfolder with the name of every analyzed image is created. This contains four files if a SCs analysis was done (Fig.19, top), or six files in case an SCs and COs analysis was executed (Fig.19, bottom), as described on the “general considerations on the operation of the macro” section.

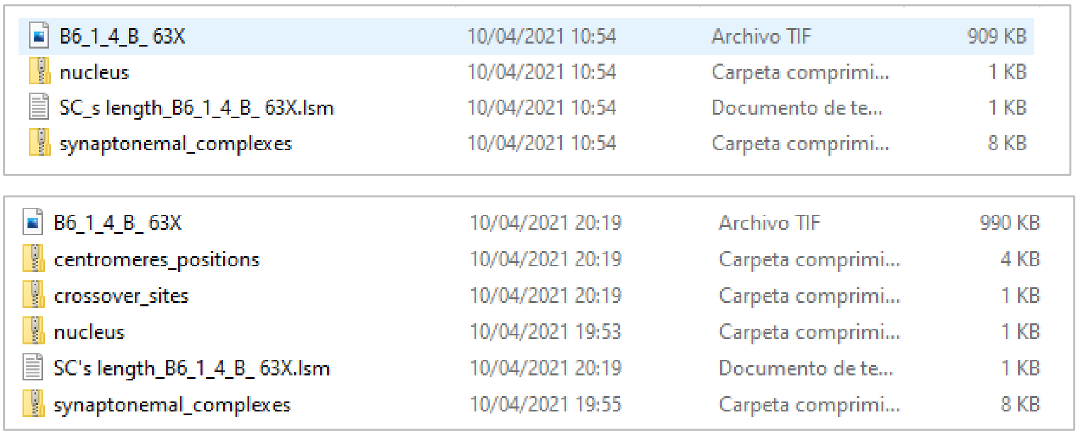

B6_1_4_B_ 63X	10/04/2021 10:54	Archivo TIF	909 KB
nucleus	10/04/2021 10:54	Carpeta comprimi...	1 KB
SC_s length_B6_1_4_B_ 63X.lsm	10/04/2021 10:54	Documento de te...	1 KB
synaptonemal_complexes	10/04/2021 10:54	Carpeta comprimi...	8 KB

B6_1_4_B_ 63X	10/04/2021 20:19	Archivo TIF	990 KB
centromeres_positions	10/04/2021 20:19	Carpeta comprimi...	4 KB
crossover_sites	10/04/2021 20:19	Carpeta comprimi...	1 KB
nucleus	10/04/2021 19:53	Carpeta comprimi...	1 KB
SC's length_B6_1_4_B_ 63X.lsm	10/04/2021 20:19	Documento de te...	1 KB
synaptonemal_complexes	10/04/2021 19:55	Carpeta comprimi...	8 KB

Figure 19: Files contained on each image results folder after running a SCs analysis (top) or a SCs and COs analysis (bottom). If a SCs and COs analysis was performed and a nucleus channel was introduced, centromere positions are recorded in the .zip file, if no DAPI channel was selected, this file would be named starting points and contain the end of each SC from which COs distances are computed.

To view the obtained results, drag and drop all files in Fig.19 but the text one (unzipped files and image) to FIJIs menu bar. This will open the image and detected SCs, nucleus and COs on the ROI Manager (Fig.20). You can visualize individual element by clicking on it in the ROI Manager or show them all by selecting the “Show all” box (Fig.21, right) The results table containing all measurements (Fig.20, center) can be opened by selecting File->Import->results command on the FIJI’s main menu and then choosing the .lsm file in Figure 19. These will be shown as shown in figure 16, and figure 2. The number of decimals of the results can be selected in Analyze> Set Measurements> Decimal Places (0-9).

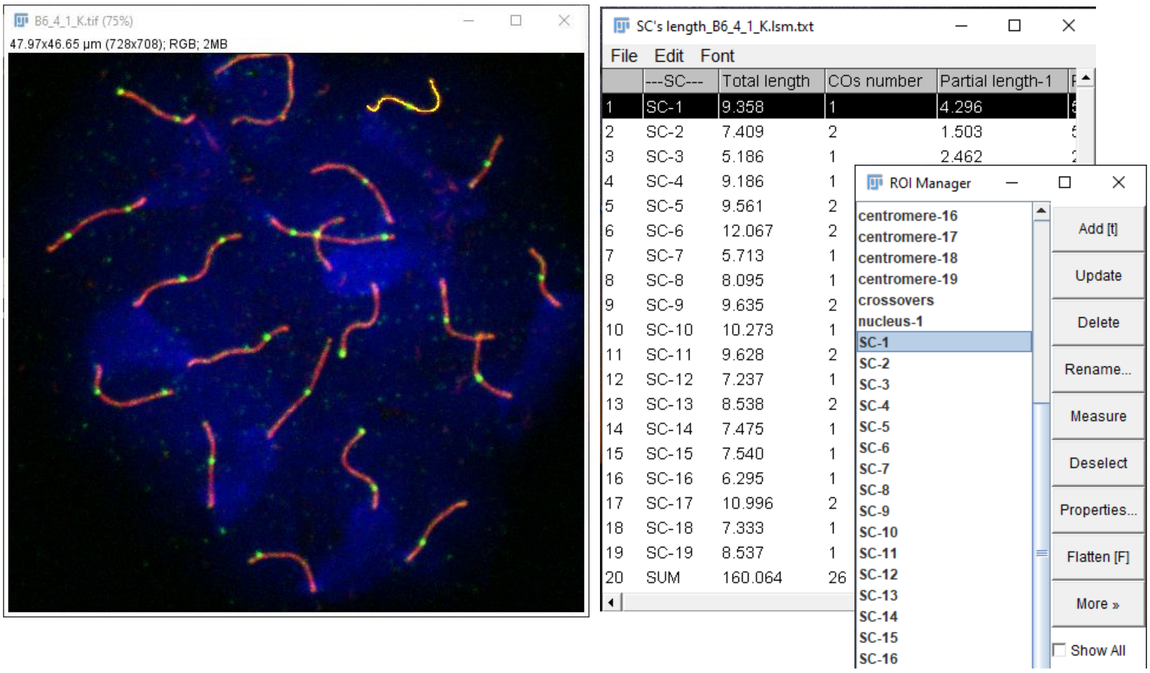

Figure 20: Analysis results as displayed in FIJI. Left: RGB image, showing the SC selected in the ROI Manager tool (right) and the table containing SC lengths (center). Zip files containing centromeres, COs and SCs can be opened in the ROI Manager and selected for visualization.

The table results after an analysis are presented as shown on Figure 20. The first column of the table is used to locate the SC in the image analyzed. The following show the total length of each SC and the total number of COs per SC (the sum of the SC lengths and the total number of COs are shown at the bottom). Partial lengths represent SC distances starting from one SC end to the first CO, from this to the next CO (if applicable) and so on, and from the last CO to the opposite SC end. The starting point of each SC measurement is the upper one by default. In telocentric organisms (such as most house mice), DAPI labeling allows to identify the centromeric end at one extreme of each SC, which become the starting point for SC length measures.

4.1. Importing results to Excel

Open excel and create a new blank workout. Go to the main Menu and click on data, get external data and select the option "from text" (figure 21, top).

Select the results text file on the pop-up window and click on import (Fig.21, bottom).

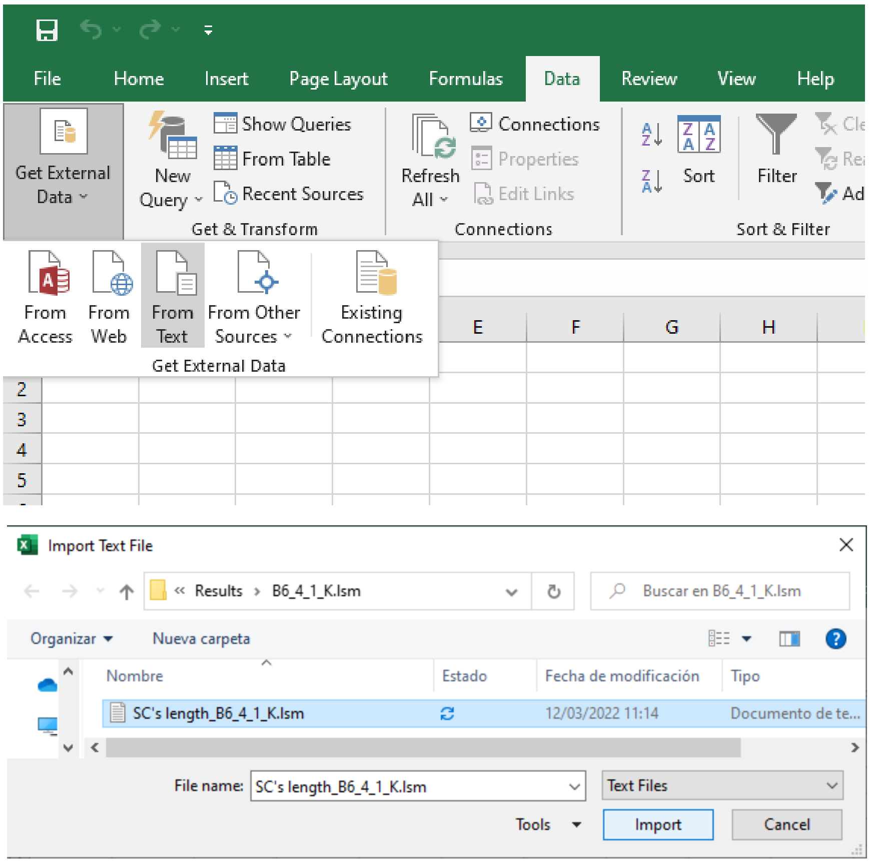

Figure 21: Opening the Text Import Wizard (top) and selecting the file to import (bottom).

Choose the option "Delimited" on the Text Import Wizard and click on Next (Fig.22, top). Select the Tab delimiter (Fig.22, middle), the General column data format and use the Advanced… option to set the decimal separator to "." and the thousand separator to blank (Fig.22 bottom). Click on OK and Finish.

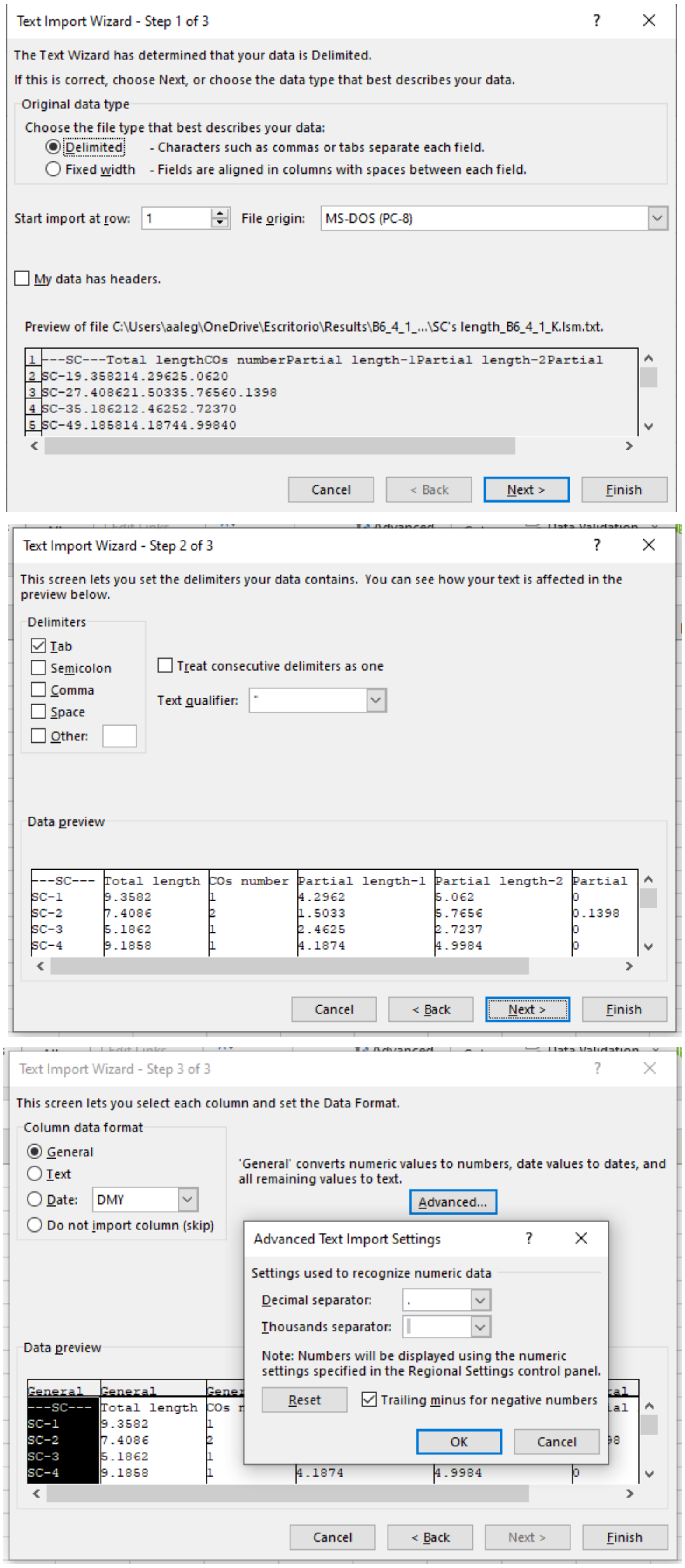

Figure 23: setting import parameters.

5. WHATS NEW ON SYNAPTONEMAL & CO ANALYZER 2.0?

5.1 SYNAPTONEMAL & CO ANALYZER 2.0 opens a wider variety of image formats

The previous version relied on imageJ's open command, which Is restricted to TIFF, GIF, JPEG, BMP, PNG, PGM, FITS and ASCII. Version 2.0 uses the Bio-Formats Importer plugin to open many dozens of proprietary life sciences formats. Bioformats is an initiative of The Open Microscopy Environment (OME), a consortium of universities, research labs, industry and developers producing open-source software and format standards for microscopy data.

More information about the Bio-Formats Importer plugin and OME can be found in the following links: https://imagej.net/formats/bio-formats and https://www.openmicroscopy.org/.

The Bioformats Importer plugin is already installed in FIJI. ImageJ users need to install it by following the instructions in this link: https://docs.openmicroscopy.org/bio-formats/6.10.1/users/imagej/installing.html.

Please notice that the Bio-Formats Importer plugin's console pops-up every time an image is opened, but can be closed or minimized if desired (Fig.24).

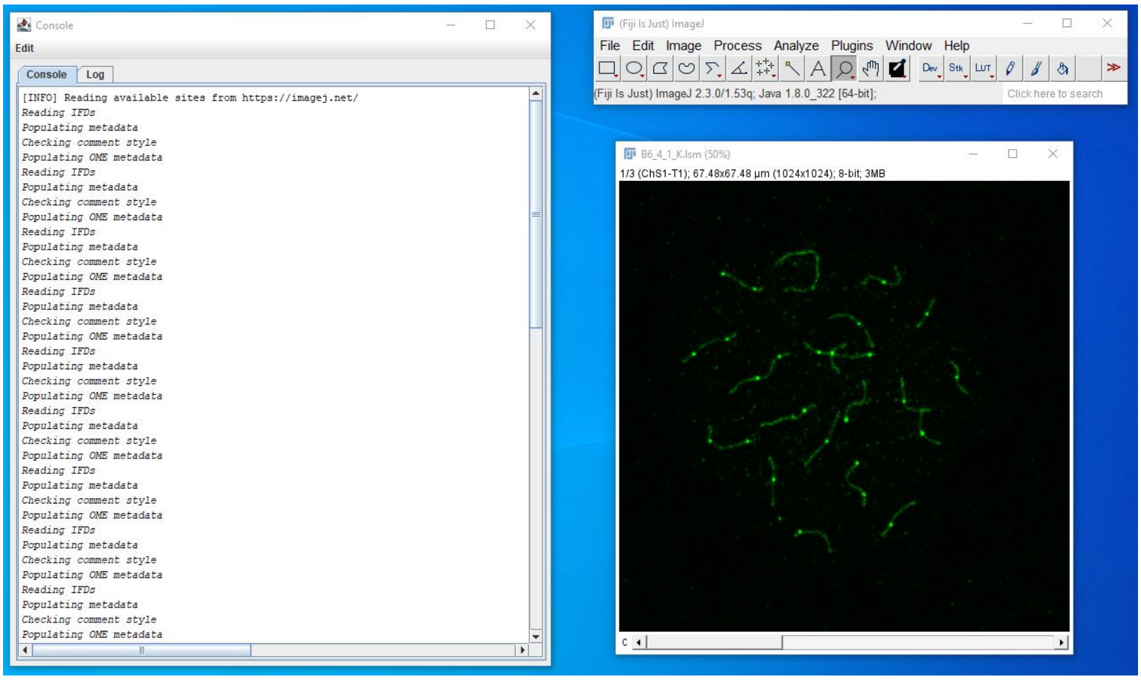

Figure 24: The console (left) is opened by Bioformats Importer plugin. It can be closed or minimized.

5.2 SYNAPTONEMAL & CO ANALYZER 2.0 eases adapting the macro to your own images

Synaptonemal & CO analyzer was initially designed to detect SCs, COs and centromeres on mouse pachytene spermatocytes, labelled with DAPI and antibodies against MLH1 and SYCP3, and captured under a confocal microscope. Therefore, the macro detection procedures installed by default might not work well on different experimental designs and conditions. There are three ways to adjust Synaptonemal & CO analyzer to your data, depending on your knowledge of image processing:

5.2.1 Users with neither imageJ programming nor image analysis skills.

SC DETECTION

Download, install and run the macro skeletonize_SC_macro_recorder.ijm (visit: 1. GETTING STARTED: INSTALLING, UPDATING AND RUNNING THE PLUGIN in this manual for further information on how to install and run a macro).

Once done, an image of your SCs will open and a series of pop-up menus will guide you through a wizard. Follow instructions on every step bearing in mind that the final goal is to get an image of the SCs' skeletons. This image does not need to be perfect since Synaptonemal & CO analyzer provides means for correcting discontinuities, split overlapping SCs, etc. (check section 3. WORKING WITH SYNAPTONEMAL & CO ANALYZER: ANALYSIS OF SCs and COs. in this manual). At some point, you will be asked whether you are satisfied with the detected SCs; you might need to find the best combination of variables -the wizard will start again in case you are not satisfied with results. Once the SC detection is good enough, the macro will end and create a file containing a piece of code (Fig.25)

Open that file, copy the code, visualize the Synaptonemal & CO Analyzer macro code by dragging and dropping it into imageJ main tool bar, use the Find and Replace command (Find/Replace…, under the Edit menu) and look for "function SC_analysis(){". Get rid of all code between brackets, paste the code you copied previously, save changes (File ->Save) and restart FIJI. Next time you run the macro, your SCs will be smoothly detected (Fig.25).

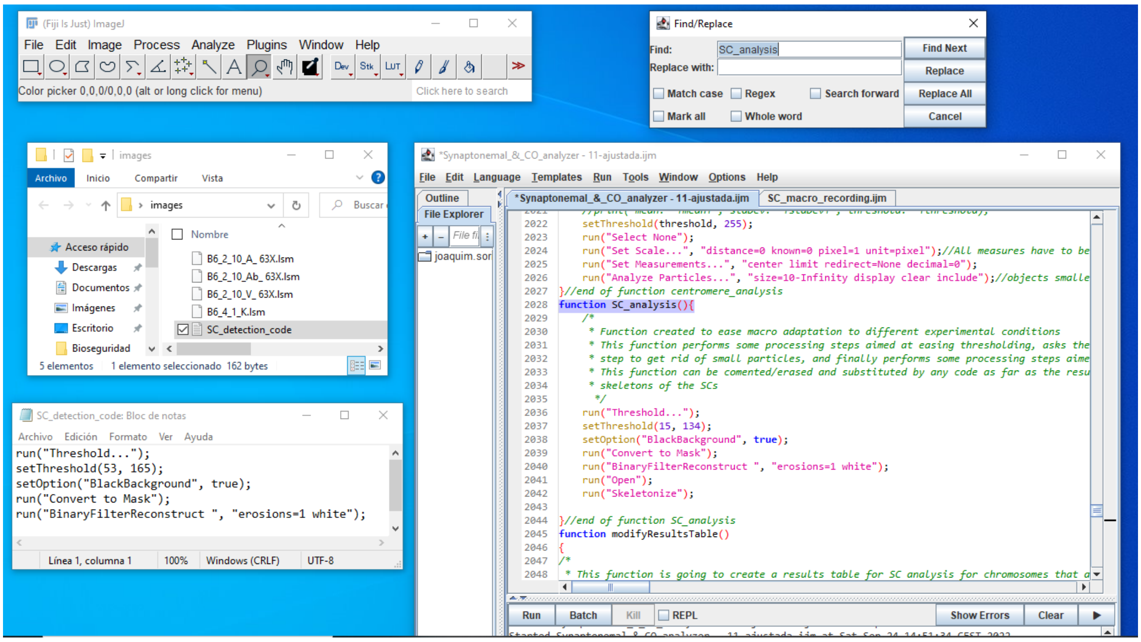

Figure 25: Synaptonemal & CO analyzer (bottom right) macro as seen when it is dragged and dropped into FIJI's main tool bar (top left). Running skeletonize_SC_macro_recorder.ijm, creates a text file on your images folder (middle left) containing a piece of code (bottom left). Use the Find/Replace command (top right) to look for the function SC_analysis in the macro (highlighted in bottom right), change it for the previously copied code in order to tuned the macro to detect the SCs in your images.

Please notice that all the code in brackets after the function SC_Analysis that is not green is the one that you need to replace (brackets should not be removed).

FOCI DETECTION

Download, install and run the macro foci_detection_macro_recorder.ijm (visit: 1. GETTING STARTED: INSTALLING, UPDATING AND RUNNING THE PLUGIN in this manual for further information on how to install and run a macro).

Once done, an image of your foci-labelled structures (usually, but not necessarily, COs or centromeres) will open and a series of pop-up menus will guide you through a wizard. Follow instructions on every step bearing in mind that the final goal is to detect labelled puncta structures. The detection does not need to be perfect since Synaptonemal & CO analyzer provides means for adding or deleting missed or wrongly detected structures.

Two basic detection algorithms are provided:

1- Based on signal brightness, size and shape: groups of pixels of a certain intensity, size and circularity range will be considered for analysis. This detection algorithm is based on the Threshold and Analize particles imageJ funtions, please visit https://imagej.nih.gov/ij/docs/menus/analyze.html - ap for details.

2- Based on local maxima analysis: pixels outstanding more than a certain intensity level from the surroundings will be considered for analysis. This detection algorithm is based on the Find Maxima imageJ function, please visit https://imagej.nih.gov/ij/docs/menus/process.html - find-maxima for details.

The macro works similarly to the skeletonize_SC_macro_recorder.ijm (see previous section). The piece of code generated should be pasted in the functions centromere_analysis() or CO_analysis() in the Synaptonemal & CO Analyzer macro, depending on whether the code was meant to detect COs or centromeres.

5.2.2 Users with image analysis experience.

Open the image channel containing the objects to detect (mainly SCs, COs or centromeres).

Click on Plugins->Macros->Record… in FIJI’s main tool bar to open the Macro Recorder (follow this link for more information on the macro recorder: https://imagej.nih.gov/ij/docs/menus/plugins.html - macros). Useyour ImageJ skills to get an appropriate binary image of SC’s skeletons or a results table containing foci (mainly COs or centromeres) centers of mass. Such table should equal the ones that imageJ creates when analyzing particles: it should contain detected foci in rows, and X and Y centers of mass coordinates in two columns entitled XM and YM. The easiest way to create this table is by performing a center of mass analysis in imageJ. In order to do so, click on “Analyze” in imageJ main tool bar, “set measurements” and select “Center of mass”. Afterwards click on “Analyze particles” under the “Analyze” menu (follow this link for further information: https://imagej.nih.gov/ij/docs/menus/analyze.html). Notice that, while processing your images, all steps you perform are saved as a macro code in the macro recorder (Fig.26).

Once SCs detection is good enough, copy and paste the code in the macro recorder to the Synaptonemal & CO Analyzer macro and proceed as explained in the previous section.

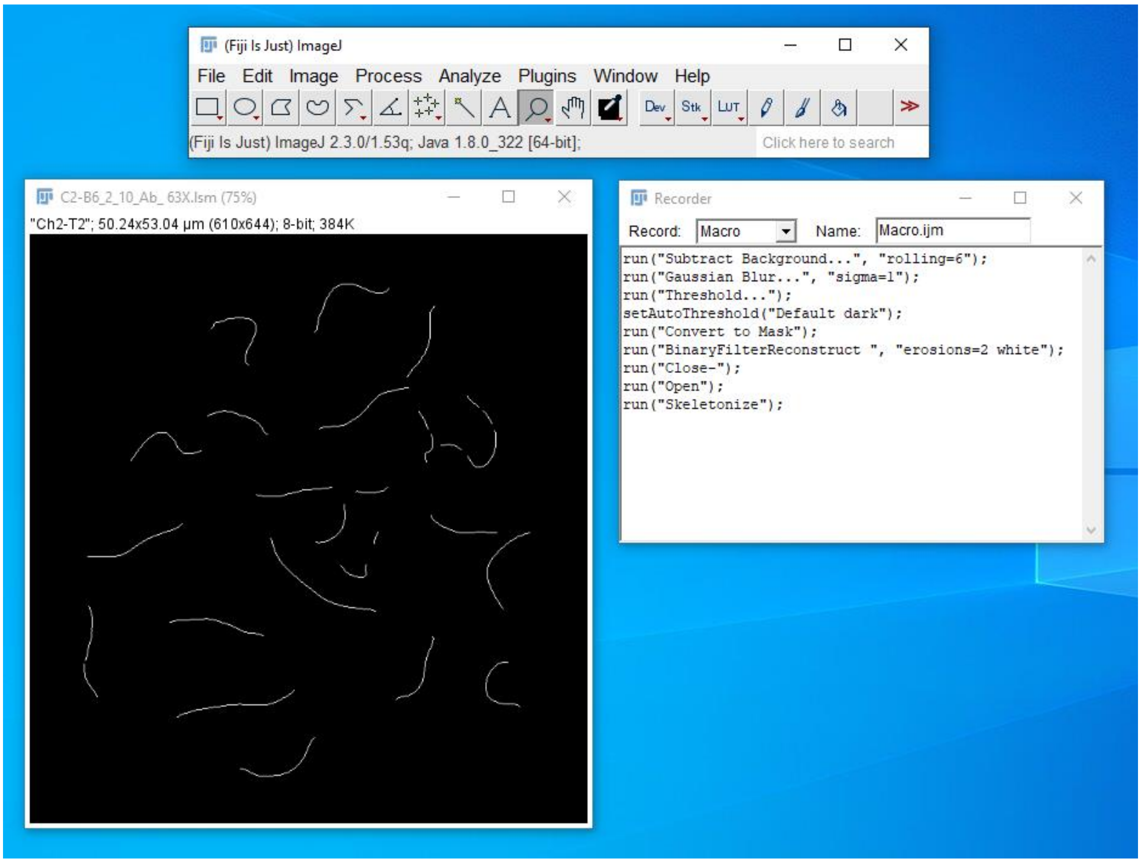

Figure 26: SCs skeletons (bottom left) and the resulting code as shown in the macro recorder (bottom right). Notice that some SCs' skeletons show discontinuities that can be corrected using Synaptonemal & CO Analyzer macro afterwards.

5.2.3 Users with imageJ macro language programming skills.

Open the script editor (Fig.25, down right) by clicking File->New->Script… in FIJI's main tool bar.

Click Language->ImageJ1 Macro in the script editor menu.

Create a script to get a binary image of SCs' skeletons or a results table containing foci (mainly COs or centromeres) centers of mass.

Copy the code into Synaptonemal & CO Analyzer macro as explained in the sections above.

5.3 SYNAPTONEMAL & CO ANALYZER 2.0 is able to analyze images with centromere-specific labeling

Synaptonemal & CO Analyzer has been modified to allow for analyzing images that detect centromeres based on a specific labelling (such as CREST serum). Although Synaptonemal & CO Analyzer 2.0 appearance is quite similar to version 1.0, you will notice that:

1- The graphic user interface shown in Fig.6 (version 1.0), now includes the option of detecting a centromeres' labelled image channel (Fig.27)
2- Later on, you will be asked to locate centromeres either manually or automatically. This step is identical to the COs identification one (as seen in section 3. WORKING WITH SYNAPTONEMAL & CO ANALYZER: ANALYSIS OF SCs and COs. of this manual and Figs.14-18).

3- Once the macro is done, results (Fig.20) will also include:
   a. Identified centromeres (Fig.28)
   b. Lengths between SCs ends and COs relative to the centromere (Fig.29)
   c. Number of COs per chromosome arm (Fig.29)

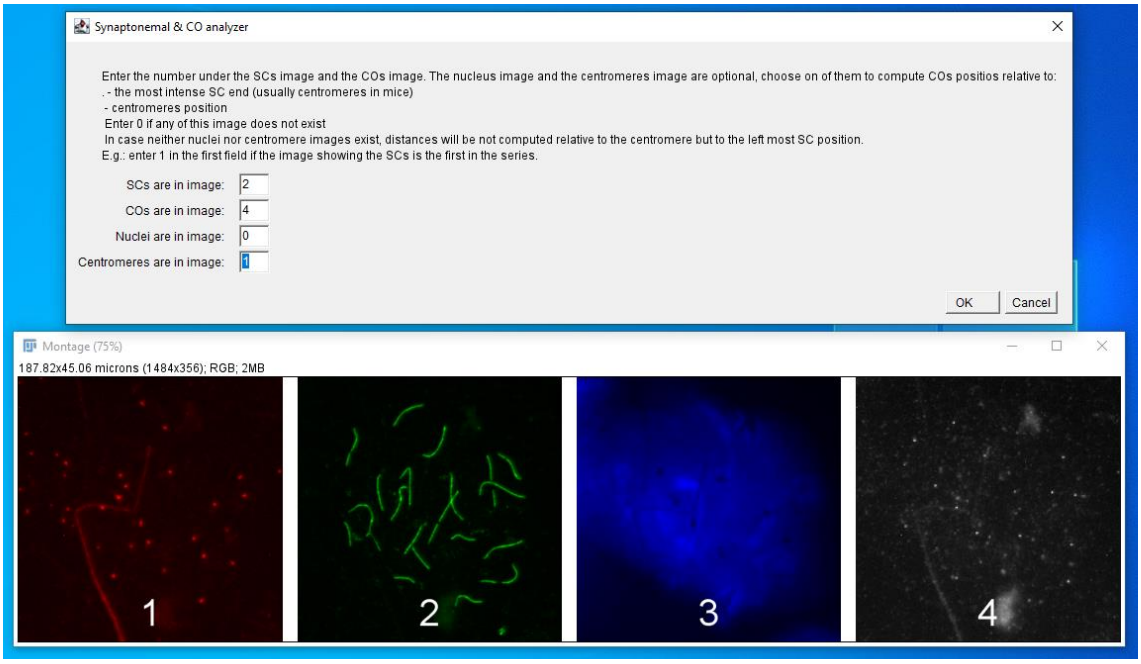

Figure 27: GUI interface of an image showing centromeres, SCs, nuclear and COs stainings. The macro will execute a SC and CO analysis respected to the position of specifically-stained centromeres if "Centromeres are in image:" field is filled, or nuclear staining centromere analysis respected to a DAPI-based centromere location if the "Nuclei are in image field:" is filled.

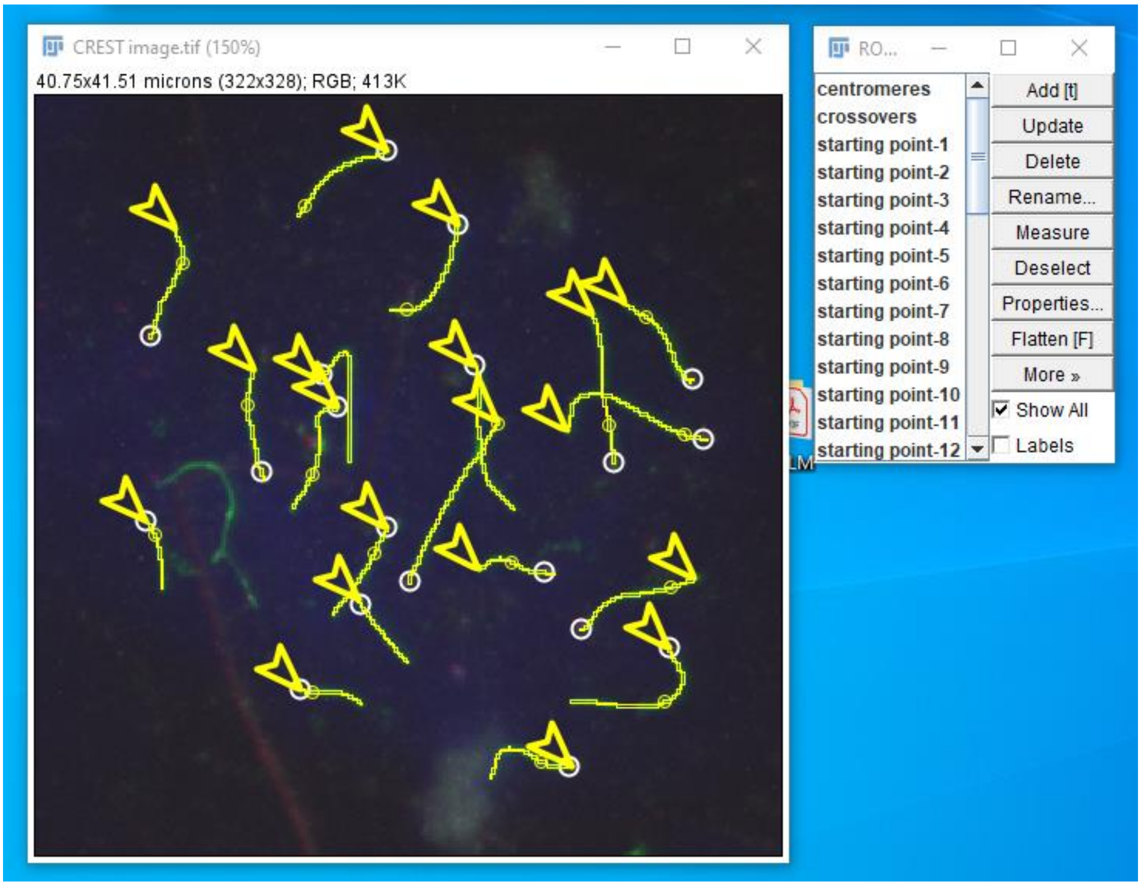

Figure 28: Results image after a specific centromere labelling analysis (left). SCs (yellow lines), COs (yellow small circles), centromeres (white big circles) and a reference point in each SC (arrow heads) can be highlighted by using the ROI Manager (right).

Results

File Edit Font Results

	---SC---	Centromere position	CO number arm 1	CO number arm 2	arm1: partial length1	arm1: partial length2	arm1: partial length3	arm2: partial length1	arm2: partial length2
14	SC-14	0.063	0	1	-0.063	0.000	0	0.685	3.009
15	SC-15	0.190	0	1	-0.190	0.000	0	1.624	4.363
16	SC-16	10.788	1	0	-0.190	-10.598	0	0.190	0.000
17	SC-17	0.089	0	0	-0.089	0.000	0	9.037	0.000
18	SC-18	10.085	1	0	-8.968	-1.117	0	0.190	0.000
19	SC-19	8.635	1	0	-6.505	-2.130	0	0.063	0.000
20	SUM	----	0	0	0.000	0.000	0	0.000	0.000

Figure 29: Results table after a specific centromere analysis. Centromere position is given relative to the reference point shown in Fig.28. Partial lengths starting on the closer end to the centromere. For distinction its values are preceded by a negative sign. Please notice that general results (as shown in Fig.20) have been removed in order to ease reading.

The results after an analysis using centromere-specific labeling are presented as shown on Figure 29. In addition to the results shown in Figures 2 and 20, when CREST serum is used to locate centromeres position, the macro provides additional data for each of the two chromosome arms separated by them. Partial length measurements start from the upper side of the SC (reference point) up to the centromere (arm 1, in negative values), from which arm 2 measurements (positive values) begin up to the other extreme. SC lengths are shown in µm when analyzed image files contain the metadata, in pixels when lost.

Please notice that detecting centromeres based on DAPI staining is still possible by following the procedure on section 5.3 SYNAPTONEMAL & CO ANALYZER 2.0 is able to analyze centromeres' labelled images in this manual (Fig.27).

5.4 SYNAPTONEMAL & CO ANALYZER 2.0 eases manual COs and centromeres detection and checking automatically detected results

The macro requires that each centromere and CO associate to a SC. When the image capture does not provide a clear overlap, the software requires ascribing them manually to the closest SC, which was challenging in the previous version in absence of an SC staining image (check section 5.3 SYNAPTONEMAL & CO ANALYZER 2.0 is able to analyze images with centromere-specific labeling in this manual).

To improve this step, we have included the option of opening a multichannel view of the image under analysis, thus facilitating the CO or centromere relocation to the correct SC. In order to use this function, simply press the "c" key during the CO or centromere correction step (Fig.30).

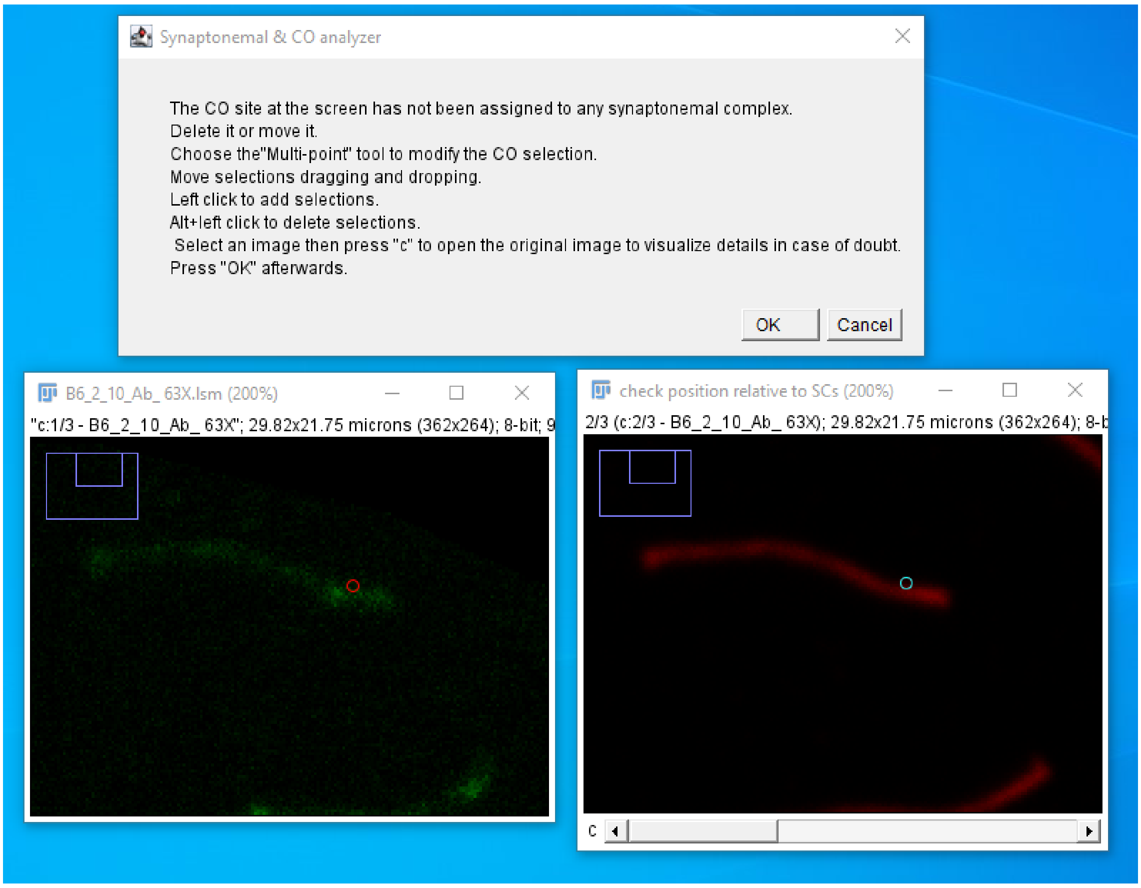

Figure 30: The CO in the image was manually misplaced. During the correction step the macro could not assign it to a SC and asked the user to so. After clicking "c" the image on the right was shown to ease for CO relocation. Notice that the bar under this image allows for selecting any channel in it.
